# Detailed tandem repeat allele profiling in 1,027 long-read genomes reveals genome-wide patterns of pathogenicity

**DOI:** 10.1101/2025.01.06.631535

**Published:** 2025-01-20

**Authors:** Matt C. Danzi, Isaac R. L. Xu, Sarah Fazal, Egor Dolzhenko, David Pellerin, Ben Weisburd, Chloe Reuter, Jacinda Sampson, Chiara Folland, Matthew Wheeler, Anne O’Donnell-Luria, Stefan Wuchty, Gianina Ravenscroft, Michael A. Eberle, All of Us Research Program Long Read Working Group, Stephan Zuchner

**Affiliations:** 1 Dr. John T. Macdonald Foundation Department of Human Genetics and John P. Hussman Institute for Human Genomics, University of Miami Miller School of Medicine, Miami, FL, USA; 2 Pacific Biosciences, Menlo Park, CA, USA; 3 Program in Medical and Population Genetics, Broad Institute of MIT and Harvard, Cambridge, MA, USA; 4 Center for Genomic Medicine, Massachusetts General Hospital, Harvard Medical School, Boston, MA, USA; 5 Stanford Center for Undiagnosed Diseases, Stanford University, Stanford, CA, USA; 6 Division of Cardiovascular Medicine, Department of Medicine, Stanford University School of Medicine, Stanford, CA, USA; 7 Centre for Medical Research University of Western Australia and Harry Perkins Institute of Medical Research, Perth, Western Australia, Australia; 8 Division of Genetics and Genomics, Boston Children’s Hospital, Harvard Medical School, Boston, MA, USA; 9 Departments of Biology and Computer Science, University of Miami, Miami, FL, USA; 10 Sylvester Comprehensive Cancer Center, University of Miami Miller School of Medicine, Miami, FL, USA

## Abstract

Tandem repeats are a highly polymorphic class of genomic variation that play causal roles in rare diseases but are notoriously difficult to sequence using short-read techniques^1,2^. Most previous studies profiling tandem repeats genome-wide have reduced the description of each locus to the singular value of the length of the entire repetitive locus^3,4^. Here we introduce a comprehensive database of 3.6 billion tandem repeat allele sequences from over one thousand individuals using HiFi long-read sequencing. We show that the previously identified pathogenic loci are among the most variable tandem repeat loci in the genome, when incorporating nucleotide resolution sequence content to measure the longest pure motif segment. More broadly, we introduce a novel measure, ‘tandem repeat constraint’, that assists in distinguishing potentially pathogenic from benign loci. Our approach of measuring variation as ‘the length of the longest pure segment’ successfully prioritizes pathogenic repeats within their previously published linkage regions. We also present evidence for two novel pathogenic repeat expansion candidates. In summary, this analysis significantly clarifies the potential for short tandem repeat pathogenicity at over 1.7 million tandem repeat loci and will aid the identification of disease-causing repeat expansions.

## Main Text

Since the initial discovery in 1991 of an expanded CGG repeat in the *FMR1* gene as the underlying cause of Fragile X syndrome, over 60 disorders have been associated with tandem repeat (TR) expansions^1,2,5^. Only recently, genome-wide exploration of TR loci has been enabled by specialized analysis of short-read sequencing data^3,4^. While this has unlocked many opportunities, fundamental technical limitations require simplification of TR variation at a given locus to a single measure: the numeric length of the repetitive region. Long-read sequencing, capable of capturing the population-level complexity of all TR loci at base-pair resolution, will be essential for advancing our understanding of TR biology and identifying medically relevant TRs.

### Length and motif variation among 3 billion tandem repeat alleles

**Figure 1:**
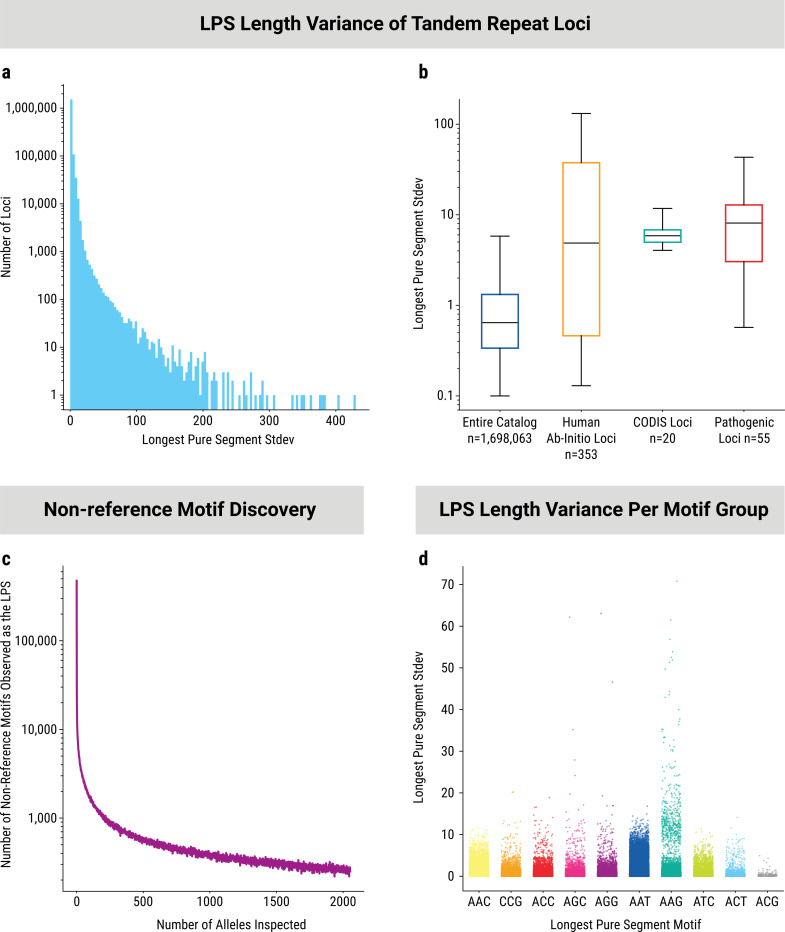
Length and motif variation among 3 billion tandem repeat alleles. A) Histogram of standard deviation of LPS length for each of the 1.7 million analyzed TR loci. B) Boxplots of standard deviation of LPS length for the entire catalog, the set of loci identified by Sulovari and colleagues to be human-specific (human ab-initio loci), CODIS loci, and the known pathogenic loci. C) Saturation curve of novel LPS motif discovery showing number of previously unseen novel LPS motifs observed per individual as individuals are added to the cohort. D) Standard deviation of LPS length for each of the 3bp simplified motif classes. Each dot represents one TR locus. For each locus, only the most commonly observed LPS motif is used. Corresponding plots for 2bp, 4bp, 5bp, and 6bp motif classes are shown in Supplementary Figures 5 and 6.

The *All of Us* Program Long Read Working Group performed HiFi sequencing on 1,027 self-reported black or African-American individuals enrolled in the *All of Us* Research Program. This manuscript serves as a TR-focused companion to the main paper by Garimella and colleagues describing the cohort and findings more broadly. We used TRGT^6^, configured with the Adotto catalog of over 1.7 million TR loci^7^, on these 1,027 long-read samples to produce a call set of 3,603,764,724 TR alleles. For each allele, TRGT generated the consensus sequence of the TR along with 25 bp of flanking sequence on each side. This form of output at this scale provides tremendous opportunities for novel analyses of the interplay between length, motif, and flanking variation at TRs across the genome^8^. The overall pattern of length variation across the 1.7 million TR loci is presented in Supplementary Figure 1a, using allele length regardless of interruptions or motif changes. This shows a large range of length polymorphisms spanning nearly four orders of magnitude. In this work, we sought to combine information about TR length and sequence content into a more informative measure. Therefore, we calculated the length of the longest pure segment (LPS) and its concomitant motif for each allele. The histogram of the pattern of LPS length variation across all the TR loci shows a clear power law distribution with a small fraction of TRs being much more variable than the vast majority (Figure 1a). While nearly all the TR loci in this catalog demonstrate some level of length variation (1,766,496 / 1,766,515; 99.999%), we observed that TR loci detected by Sulovari and colleagues^9^ as non-repetitive in Great Apes (human ab-initio loci) exhibit significantly greater LPS length variance than the catalog as a whole (Mann-Whitney U Test: U=420,104,161.5, p=4.67e-39) (Figure 1b), suggesting that evolutionary recency increases the extent of variation in tandem repeats. Furthermore, we observe that known pathogenic loci exhibit significantly greater levels of LPS length variance in this control population compared to the catalog (Mann-Whitney U Test: U= 82,509,469.5.5, p= 6.67e-23) (Figure 1b). However, this result is not specific enough to proactively identify novel pathogenic loci, delineating them from other hyper-polymorphic loci that are unlikely to be pathogenic, such as the twenty CODIS loci used in forensic analyses. Additional genome-wide patterns of TR length variation are discussed in the Supplementary Results and Supplementary Figure 1. For known pathogenic loci, the distribution of allele lengths observed is provided in Supplementary Table 1 and the distribution of LPS lengths observed is provided in Supplementary Table 2.

We next used the motif associated with each LPS measurement for the unbiased identification of novel motifs within TRs. A saturation analysis with this approach revealed that even after examination of 1,027 genomes, approximately 260 novel LPS motifs were observed with each additional sample added to the set (Figure 1c). This indicates that many more samples need to be analyzed to observe all possible LPS motifs. Comprehensive characterization of novel LPS motif occurrence rate and how these changes affect the period of repetition are presented in the Supplementary Results and Supplementary Figures 2–4.

Next, we compared groups of TRs stratified by “simplified LPS motif” using standard deviation of LPS length. In this schema, motifs that are equivalent get grouped together (see methods for details). We observed marked differences among the ranges and distributions of values observed for different simplified motif groups which are driven by a relatively small percentage of the loci within each group (Figure 1d, Supplementary Figures 5–6). Interestingly, motifs already associated with tandem repeat disorders such as GAA (FXN and SCA27B; represented as AAG in the plot), and CAG (polyglutamine disorders; represented as AGC in the plot) contained some of the most variable loci. This runs counter to the expectation of generally high constraint of variation in controls at disease loci. For the GAA, CAG, and AGG motifs, we observed multiple loci with standard deviation values in the range of 30 to 70, while the other seven simplified trinucleotide motifs do not have any loci with values over 20.3 (Figure 1d).

### Pathogenic loci are among the most variable TRs in the genome for each motif

**Figure 2:**
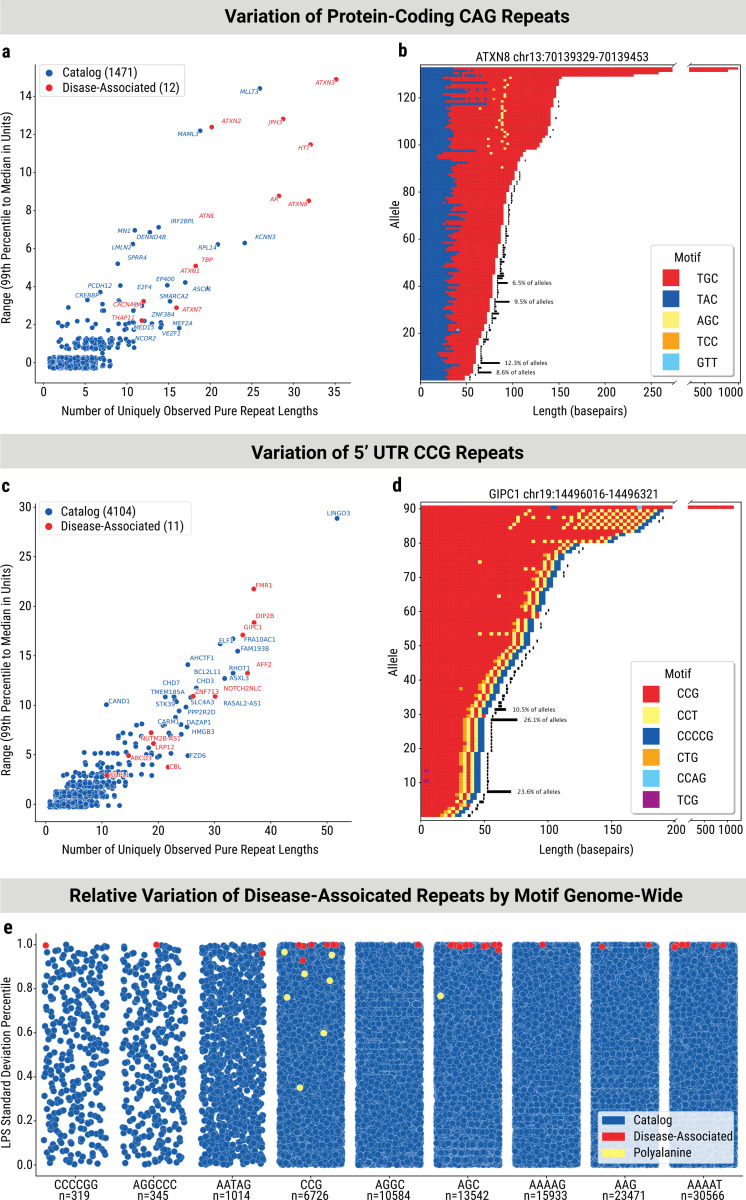
Variation at known pathogenic TRs in healthy individuals. a) Scatterplot of the number of unique repeat lengths and pure CAG LPS range (99^th^ percentile to median) at protein-coding CAG loci. Disease-associated loci are shown in red and catalogued loci are shown in blue. The 20 most variable catalog loci are labelled with their gene name. c) Scatterplot of the number of unique repeat lengths and pure CGG LPS range (99^th^ percentile to median) at 5’ UTR CGG loci. Disease-associated loci are shown in red and catalogued loci are shown in blue. The 20 most variable catalog loci are labelled with their gene name. b, d) Waterfall plots of repeat alleles for ATXN8 (b), and GIPC1(d). All alleles observed at least 3 times were plotted in conjunction with the largest 1% of alleles. Alleles are sorted by decreasing length. Normalized frequency of observed alleles is shown in black following each segmentation. e) Percentile of LPS length standard deviation at disease associated TR loci with one strip for each relevant simplified motif. For each locus, the most common (wildtype) motif seen at the locus is used. The height is the percentile from 0–1. Catalog points are in blue, while polyalanine disease-associated loci are in yellow, and all other disease-associated loci are in red.

Protein-coding CAG repeats (often referred to as poly-glutamine repeats) and non-coding CGG repeats in the 5’ UTR of genes comprise significant portions of disease associated TRs with over 10 genes in each group. It is plausible that additional coding CAG and 5’ UTR CGG genotypes can cause disease when expanded. To delineate variational differences for these two groups, we analyzed the range of observed LPS lengths (99^th^ percentile minus median) and the number of uniquely observed pure repeat lengths in 1,483 protein-coding CAG and 4,115 CGG 5’ UTR TR loci contained within the catalog. Known pathogenic loci exhibited greater levels of variation compared to loci without established disease associations (Figure 2a,c). This analysis also highlights candidate repeat loci that emulate the notable variability seen at known pathogenic loci. These unstable loci warrant investigation for outlier genotypes, especially in patients with rare neurological diseases.

We selected one protein coding CAG and one 5’ UTR CGG locus to describe with their sequence content in detail (Figure 2b,d). Variation was observed in the quantity and sequence content of interruptions found within these repeat tracts. For instance, at both of these known pathogenic loci, we observed novel non-reference interruption patterns present in the largest alleles (Figure 2b,d). At the *FMR1* 5’ UTR CGG locus, we observed variable numbers of AGG interruptions, with intermediate alleles (45–54 repeats) containing higher proportions of 3, 4, and 5 AGG interruption alleles (Supplementary Fig 9). This level of resolution is missed using conventional short-read tools and increases our understanding of normal variation present at disease-associated and disease candidate TR loci.

To interrogate this observation further, we compared variation at known pathogenic loci to all loci genome-wide that share the same motif. We found that the standard deviation of pure repeat length at known pathogenic loci ranked among the highest for each motif class in the entire genome, even when including intergenic repeats (Figure 2e). Notably, in our cohort, 70.8% of all known pathogenic TRs surpassed the 99^th^ percentile or higher in variability of the LPS length within known motif groups. The effect size (Cohen’s d) of the difference in LPS standard deviation between known pathogenic and all other TRs was very large at 2.97.

Pathogenic loci with pentanucleotide motif AAAAT/TTTTA repeats (e.g., BEAN1, DAB1, STARD7) all require a motif change or insertion to cause their respective diseases. That is, AAAAT/TTTTA repeats alone are benign at any length in these genes, as far as is currently understood^10–12^. Despite this, the AAAAT repeats in these genes are still among the top 1 percentile of length variance, highlighting the instability of these loci compared to other AAAAT loci in the human genome. This is particularly surprising, as AAAAT loci had some of the largest values for variation of any of the motif classes inspected (Supplementary Figures 5–6). Similarly, although AAAAG repeats at the RFC1 locus are considered benign, this locus is the second most variable AAAAG repeat locus genome-wide in our population.

Furthermore, the coding CAG repeats are all within the top 1 percentile of CAG length variance genome wide. In theory, intergenic and intronic CAG TRs should be under less mutational constraint, yet these results show that the known pathogenic coding CAG loci exhibit exceptional instability for that motif even within benign ranges. This pattern of hyper-polymorphism held true for all known pathogenic TRs except for the group of coding poly-alanine repeats (HOXD13, NIPA1, ZIC2, HOXA13, PHOX2B, RUNX2, ARX, SOX3, PABPN1), shown in yellow in Figure 2e. These TRs are composed of imperfect (low purity) GCN repeats and have relatively low pathogenic expansion thresholds.

Studying the 99^th^ percentile versus median LPS TR length genome-wide also effectively highlights the instability at pathogenic loci (Supplementary Figure 7). For example, the CCGGGG repeat in C9orf72 exhibited the largest difference between the median and 99^th^ percentile length for its motif group. It is important to note that these differences are within benign ranges (the 99^th^ percentile being 13 CCGGGG units) and consider all 319 CCGGGG repeats genome-wide. While the addition of more ancestral groups and control genomes that contribute rare alleles may change the exact percentile rankings, we expect this observation to hold true: in the general population, the variation in LPS length at pathogenic TR loci is among the highest of all TR loci sharing the same motif.

### Disease-causing TRs can be readily identified from linkage region and GWAS data

**Figure 3:**
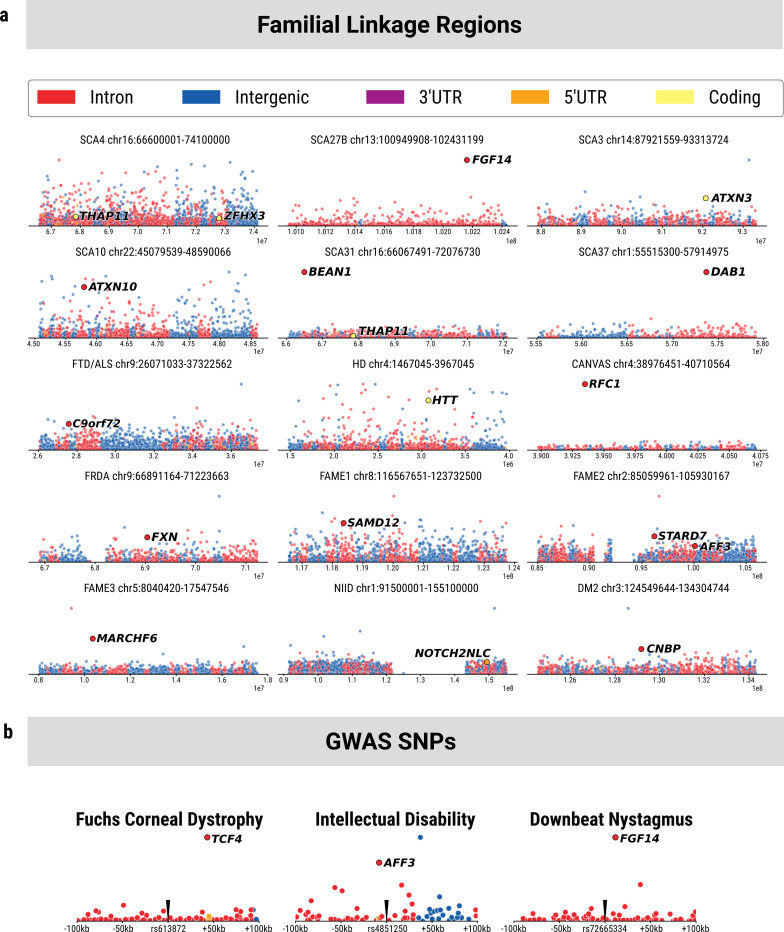
Re-identification of pathogenic TRs from published linkage regions and GWAS data. a) Series of Manhattan plots of TRs across 15 published linkage regions. In each panel, the x-axis is the genomic coordinates across the linkage region span and the y-axis is the LPS length standard deviation of each TR using its most common LPS motif. Known pathogenic TRs are specifically labelled in each plot. b) Manhattan plots of TRs within 100kb of each of 3 published GWAS SNPs, with the associated phenotype identified above each panel. In each panel, the x-axis is the genomic distance from the identified SNP and the y-axis is the LPS length standard deviation of each TR using its most common LPS motif. Known pathogenic TRs are specifically labelled in each plot.

We extend this concept and theorize that novel disease-causing TRs will also be highly polymorphic in the general population. To test this hypothesis, we retrospectively examined published linkage regions of 15 known pathogenic loci (Figure 3a). The most recent example of this is SCA4, attributed to a GGC exonic repeat in the *ZFHX3* gene ^13–17^. The linkage region for this disorder was identified nearly 30 years prior to finding the causal TR. In this cohort, while not as variable as intergenic and intronic repeats, the GGC repeat in *ZFHX3* was the fifth most polymorphic coding repeat in the 7.5 Mb linkage region. The CAG repeat in *THAP11* was the second most polymorphic coding repeat in the region and was identified as disease-associated in 2023^18^. We repeated this analysis using the published linkage regions for 14 other disease TR loci^19–32^. In every instance, the pathogenic TR locus was amongst the 99^th^ percentile most polymorphic for that region when measured by standard deviation of LPS length for the most common LPS motif. In four of the 14 cases, the pathogenic TR was the top-ranked locus (CANVAS, SCA27B, SCA31, and SCA37). This approach, in combination with the other metrics mentioned above, could have assisted in gene discovery efforts by highlighting unstable regions of the human genome. Additionally, several disorders are still only referred to by their linkage region, as the causal gene has not yet been found. Investigation of tandem repeats in these linkage regions may find pathologically expanded loci according to our methodology.

To further explore the role of TR polymorphism in health and disease, we examined published GWAS data (Figure 3b). We illustrate three GWAS SNPs associated with phenotypes within 50kb of a tandem repeat later associated with a disease where the phenotype from the GWAS association matched symptoms of the disease. In each case, the causal TR was the first- or second-most polymorphic TR within regions of possible linkage disequilibrium with the identified SNP, measured by the standard deviation of LPS length for the most common LPS motif. While not all GWAS signals are likely explained by neighboring TRs, this analysis highlights a subset of non-coding variation that provided functional insight into these previously identified GWAS results. Notably, this underscores the idea that the causal variant driving these associations could have been identified earlier based on the high population-level variability of these TRs relative to other TRs in the region.

### *EP400* and *FAM193B* are promising candidate pathogenic tandem repeat expansions

**Figure 4:**
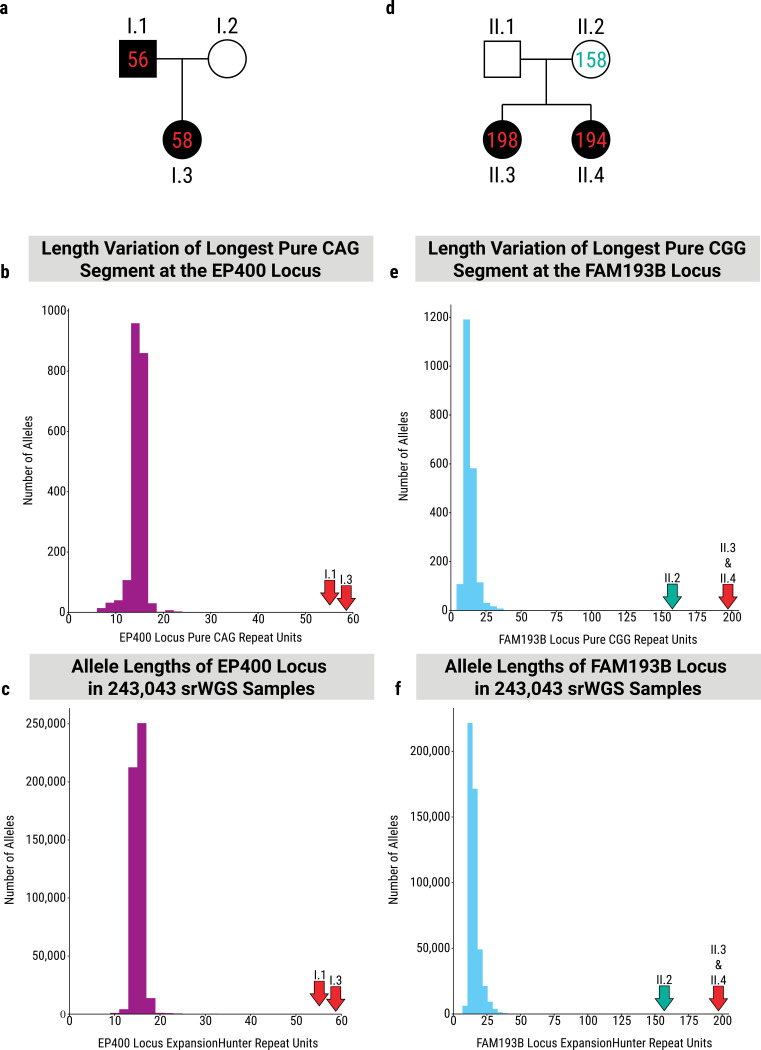
Investigation of *EP400* and *FAM193B* candidate pathogenic TR loci. a) Pedigree of the family with ataxia. Number of CAG repeats measured at the *EP400* locus is given for each measured family member. b) Histogram of LPS length for the CAG motif at the *EP400* locus in this cohort of 1,027 individuals. Red arrows mark the allele sizes observed in the two affected individuals who were not part of this cohort. c) Histogram of repeat unit count at the *EP400* locus in the *All of Us Research Project* short-read cohort of 243,043 individuals. Red arrows mark the allele sizes observed in the two affected individuals who were not part of this cohort. d) Pedigree of the family with OPDM. Number of CGG repeats measured at the *FAM193B* locus is given for each measured family member. e) Histogram of LPS length for the CGG motif at the *FAM193B* locus in this cohort of 1,027 individuals. Red arrows mark the allele sizes observed in the two affected siblings who were not part of this cohort. The green arrow marks the allele size observed in the unaffected parent. f) Histogram of repeat unit count at the *FAM193B* locus in the *All of Us Research Project* short-read cohort of 243,043 individuals. Red arrows mark the allele sizes observed in the two affected siblings who were not part of this cohort. The green arrow marks the allele size observed in the unaffected parent.

Pursuant to the prediction above that disease-causing TRs are likely to be among the most polymorphic loci, we encountered a family where the father and daughter both presented with spinocerebellar ataxia at ages 42 and 43, respectively (see Supplementary Results for complete details) (Figure 4a). ExpansionHunter analysis of short-read genome sequencing data revealed the presence of an expansion in the polyglutamine tract of *EP400*. Follow-up long-read sequencing using Oxford Nanopore Technologies (ONT) instruments confirmed the expansion to contain a pure tract of 56 CAG repeats in the father and 58 CAG repeats in the daughter. This CAG repeat in *EP400* is the 18^th^ most polymorphic protein-coding CAG locus genome-wide in our dataset (median LPS TR length to the 99^th^ percentile LPS length) (Figure 2a). The longest pure CAG segment at this locus observed in our set of 1,027 controls was 24 repeats. The distribution of the lengths of the longest pure CAG segment in this cohort is presented in Figure 4b. We further genotyped the *EP400* repeat in the 243,043 individuals from the *All of Us Research Project*’s short-read genome cohort, which are presented in Figure 4c. *EP400* is highly constrained for SNV variation and is highly expressed in the cerebellum. The pattern of expression is reminiscent of several other genes that cause spinocerebellar ataxia through poly-glutamine expansions, including *ATXN2*, *ATXN3*, and *ATXN7* (Supplementary Figure 11). This poly-glutamine repeat was also predicted to be pathogenic by the RExPRT tandem repeat pathogenicity prediction AI tool with a perfect score of 1.0^33^. While we have not been able to identify any additional families carrying this expansion and present with a matching phenotype, we propose this expansion in *EP400* as a strong candidate for a novel spinocerebellar ataxia.

A second candidate locus uncovered with this approach is a CGG repeat in the 5’ UTR of the *FAM193B* gene (Figure 2c). The expansion of several other CGG repeats located in 5’ UTRs have been implicated in oculopharyngodistal myopathy (OPDM): *NOTCH2NLC*, *GIPC1*, *LRP12*, *RILPL1*, and *ABCD3*. The CGG repeat in *FAM193B* is the seventh most polymorphic 5’ UTR CGG repeat in our cohort when measured by the range of the median LPS length to the 99^th^ percentile LPS length (Figure 2c). In one family identified through the Undiagnosed Diseases Network, two siblings presented with OPDM at ages 49 and 51, while their parents were unaffected (see Supplementary Results for complete details) (Figure 4d). ONT sequencing revealed pure CGG repeats of 198 and 194 copies in the two affected siblings with a shorter tract of 158 pure CGG repeats in the unaffected mother. The father was not available for sequencing. The distribution of the lengths of the longest pure CGG segment at this locus in our set of 1,027 controls is shown in Figure 4e. We further genotyped the *FAM193B* repeat in the 243,043 individuals from the *All of Us Research Project*’s short-read genome cohort, which are presented in Figure 4f. We found one allele in our cohort with ~154 repeats, though it contained motif impurities which may have been sequencing artifacts. All other control chromosomes carried fewer than 50 repeats. This CGG repeat was also predicted to be pathogenic by RExPRT with a score of 0.997^33^. This gene was also prioritized as a strong pathogenic candidate for this particular family by Watershed-SV, a tool that incorporates expression data into structural variant prioritization models^34^. That tool predicts that the expansion causes overexpression of the *FAM193B* gene. Due to the greatly increased length of the pure CGG segment in these individuals and the phenotypic presentation that matches five other genes with similar 5’ UTR CGG expansions, we believe this locus represents another strong candidate. Ultimately, only additional OPDM patients carrying a *FAM193B* expansion will provide the needed confirmation.

### TR constraint reveals selective pressures on TR loci

Finally, we sought to integrate all that we learned about TR variation in the above analyses into a formal metric to further refine interpretation of any TR locus and aid in the discovery of future novel pathogenic TRs. Given the success of gene-level measures of constraint in prioritizing disease genes in recent years, we aimed to develop an analogous measure of constraint to prioritize novel pathogenic TRs.

**Figure 5:**
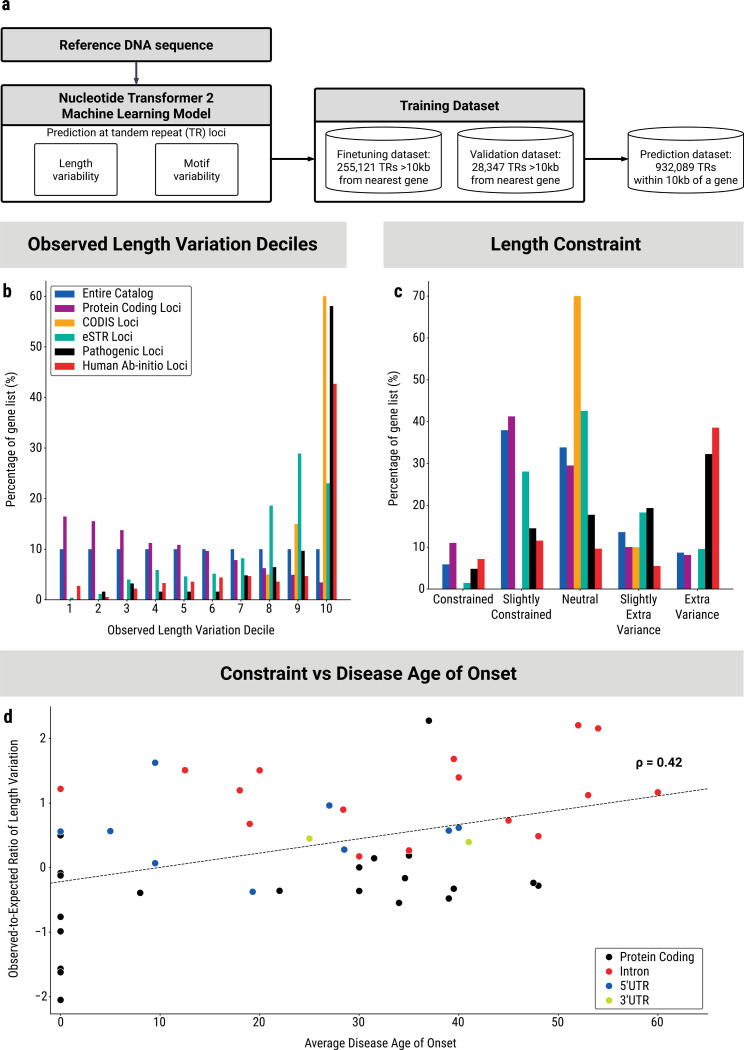
Tandem repeat constraint reveals selective pressures on TR loci. a) Schematic of the deep learning system to predict the length and motif variation of TRs based only on their reference DNA sequence by fine-tuning a 500 million parameter Nucleotide Transformer 2 model. The three datasets used to fine-tune, validate, and evaluate the constraint model are also described. b) Grouped bar plot of the percentage of isolated STRs belonging to each of the five specified sets which fall into each of the deciles of observed length variation. Observed length variation for each TR is the standard deviation of the length of the longest pure segment. c) Grouped bar plot of the percentage of isolated STRs belonging to each of the five specified sets which fall into each of the bins of length constraint. The observed-to-expected ratio of LPS length variation for each of the bins are defined as: Constrained (0–0.33), Slightly Constrained (0.33–0.75), Neutral (0.75–1.25), Slightly Extra Variance (1.25–2.0), Extra Variance (over 2.0). d) Scatterplot of the average age of onset of disease for each of 55 known pathogenic tandem repeats plotted against the logarithm of their observed-to-expected ratio of length variation (TR length constraint).

We fine-tuned a Nucleotide Transformer v2 “DNA language” model to simultaneously predict the standard deviation of LPS length and motif composition polymorphism score when given the reference sequence of a TR, padded by 50bp on each side (Figure 5a). This deep learning model was trained on TRs more than 10kb away from the nearest gene, which are presumably largely under neutral selection. Validation was performed on a held-out set comprising 10% of those loci (Figure 5a). The model achieved Spearman correlation coefficients of 0.734 for length variation (95% confidence interval: 0.728 to 0.739) and 0.682 for motif variation (95% confidence interval: 0.676 to 0.689) on the validation dataset.

The model was then used to generate ‘expected’ length and motif variation rates for all TRs within 10kb of genes. These were compared to the observed variance rates to generate an observed-to-expected ratio, analogous to gnomAD’s measure of genic constraint (Supplementary Figures 12a–b, 13a). Evaluation of these scores focused on a subset of 250,236 genic TRs, which we term ‘isolated STRs’, characterized by having only one repeat motif in the reference genome (2–6 bp) and no other identified repeating pattern within 25bp on either side. For any result shown on this subset of the data, a corresponding figure exists in the Supplementary Results, displaying the same information for the complete set of genic loci. The complete results are available in Supplementary Table 3.

We observed that, similar to non-synonymous SNVs in genes, most TRs exhibit moderate negative selection for length (observed-to-expected ratio below 1) (Supplementary Figure 12b), while an overwhelming majority of them are constrained for motif changes (Supplementary Figure 13a). Encouragingly, the set of CODIS loci, which are highly polymorphic but under neutral selection for length changes, were well-predicted by the length constraint model, achieving a Pearson correlation coefficient of 0.92 on this set of loci (Supplementary Figure 12c). The motif constraint model also performed well on the CODIS loci, achieving a Pearson correlation coefficient of 0.79 (Supplementary Figure 13b).

While direct observation of TR LPS length variation yields interesting trends for pathogenic repeats, it ultimately fails to distinguish pathogenic loci from other highly polymorphic (yet seemingly not biologically relevant) loci like the CODIS set. Both these groups of repeats fall primarily in the top 10% of most polymorphic repeats for length variation (Figure 5b, Supplementary Figure 14a). However, when using the observed-to-expected ratio based on our model, this limitation is greatly alleviated. CODIS loci are identified as under neutral selection, protein-coding TRs show less variance than expected, and pathogenic and recently evolved TRs exhibit significantly more variance than expected (Fisher’s Exact Test OR = 6.0, p = 1.5 e-8 for pathogenic loci; OR = 12.0, p = 1.8 e-76 for human ab-initio loci) (Figure 5c, Supplementary Figure 14b).

These observations demonstrate the utility of this length constraint measure and highlight a crucial distinction between TR constraint and genic constraint. With TR constraint, both tails of the distribution are biologically relevant, whereas for genic constraint, solely the loci whose variation is selected against are medically significant. Consequently, we observe that TRs at protein-coding loci are largely on the lower end of the observed length variation distribution (Figure 5b, Supplementary Figure 14a), and appear to be mostly under at least some degree of constraint (Figure 5c, Supplementary Figure 14b).

Similar to the CODIS loci, the set of gene expression-associated STR (eSTR) loci identified by Fotsing and colleagues^35^ show generally high levels of LPS variation (Figure 5b, Supplementary Figure 14a), consistent with their ability to affect gene expression. Interestingly, the constraint metric indicates that they are primarily under weak selection, forming a broad bell curve peaking in the ‘neutral’ category (Figure 5c, Supplementary Figure 14b).

We attempted to perform several orthogonal validations of the utility of this measure. First, we observed that the logarithm of TR length constraint is moderately correlated (Spearman correlation of 0.42) with the average age of onset among a set of 55 TR-associated diseases (Figure 5d). Second, we compared the TR length constraint scores with pathogenicity predictions made by RExPRT. The RExPRT AI system does not incorporate any information about length variation, instead scoring loci based on their proximity to regulatory regions and TAD boundaries, expression of nearby genes, and repeat motif composition^33^. We observe that TRs in both the extra variation and constrained bins were significantly enriched for RExPRT pathogenic predictions, while TRs in the neutral bin were significantly enriched for benign predictions (Supplementary Figure 15).

This predictive model of TR variability can also be used to perform counterfactual analyses interrogating which aspects of a nucleotide sequence result in changes in the predicted TR variability. This enables *in silico* experiments such as investigation of which motifs and motif lengths are most conducive to greater TR variability, how flanking sequence characteristics influence TR variability, and determining the precise interplay in effect between LPS length and total TR length with interruptions as they influence TR variability.

## Discussion

In this work, we have demonstrated the extensive length and motif variation present at TR loci in a large set of genomes drawn from the general population. We introduced methods and frameworks aimed at helping others interpret full sequences of these loci without reducing them to overly simplistic representations. We demonstrated that using sophisticated measures of TR variability, such as LPS length variation, can reveal clinically informative patterns. Specifically, we revealed a strong enrichment of variation in LPS length in population controls among loci whose expansion causes rare disease. Furthermore, we introduced a novel measure, ‘TR constraint’, that can assist in evaluating potentially pathogenic TRs. This measure can differentiate highly polymorphic TRs under neutral selection from those under positive selection, which includes most of the known pathogenic loci as well as human lineage-specific TR loci.

Our study has several important limitations. First, the database of allelic variation we present here is derived exclusively from individuals who have self-identified as black or African-American. As a result, there are likely some loci that are more polymorphic in other populations whose global variation is underestimated here. Future work will need to extend our described concepts to more diverse cohorts. Second, the number of individuals used here is not exceedingly large by current standards from analogous short-read studies. Future work will need to focus on significantly increasing the number of individuals analyzed in this way. Third, our study only utilizes data from PacBio HiFi genomes. While that sequencing technology is broadly accepted as being very accurate, there may be certain biases introduced by that method for particular repeat motifs that may create allele dropout or inaccuracies in our database. Future work will need to compare results produced by PacBio genomes to those produced by ONT.

Our results show divergent levels of variation for different TR motifs and replicate and extend the pioneering work of Clark and colleagues^36^. These patterns may suggest differing forces of selection on different motifs, either due to molecular utility or their potential for pathogenicity. Previous studies have observed the relationship between long, pure repetitive segments and pathogenicity^9,37^. Sulovari and colleagues suggested that loci with at least 40 pure repeats in several healthy humans would be excellent candidates for pathogenic expansions^9^. However, our study is the first to systematically show that population measures of variance in long, pure repetitive segments can reasonably differentiate known pathogenic TRs within a large TR catalog and prioritize novel loci for investigation. We believe that the loci we highlighted as novel potentially pathogenic candidates will be important to test in patients, especially, but not exclusively, with rare neurological and movement disorder phenotypes. Screening of additional individuals with spinocerebellar ataxia and OPDM will hopefully identify more families carrying the expansions we highlighted in *EP400* and *FAM193B*, respectively. Furthermore, the highly variable TR loci identified here can be viewed as a form of a reverse genetic screen, where we have isolated the variation and locus of interest and are in search of the corresponding phenotype. This stands in contrast to the classic forward genetics that starts with a phenotype of interest.

Genic constraint and local constraint measures have greatly enhanced the identification of causal SNVs and small indels in human genetics^38,39^. Here, we introduce an analogous measure for tandem repeats, which we term “TR constraint”, aimed at having a similar utility for tandem repeat evaluations. However, while the observed-to-expected (OE) ratio for genic constraint is useful for distinguishing genes with dominant effects (OE values near 0) from those with recessive effects (OE values near 0.5) and those that are dispensable (OE values near 1), TR constraint segregates TRs into those under negative selection (OE values less than 1), those under neutral selection (OE values near 1), and those under positive selection (OE values greater than 1). This difference arises from the types of observations used in the constraint models: loss-of-function and missense variants for genic constraint models versus variation in the longest pure repetitive segment and motif composition for the TR constraint models presented here. These differences in molecular mechanism and structure drive the divergent behaviors of these models, although both aim to aid in identifying the genetic causes of disease. While much of our work here focuses on the novel findings related to the hyper-variable end of this distribution, we acknowledge that numerous known pathogenic TRs exist on the constrained end of the distribution as well and we certainly expect that number to grow in the years to come.

## Methods

### Long Read Sequencing

A cohort of 1,027 individuals were selected for long-read sequencing as part of the All of Us Long Reads Working Group. All of these individuals self-identified as Black or African-American. These individuals were unrelated to each other and not known to have a rare disease. Blood samples had been collected for each of these individuals and stored in accordance with the All of Us Research Project protocols. High molecular weight DNA was extracted and sent to the HudsonAlpha Institute for Biotechnology (Huntsville, AL) for sequencing. Samples were sequenced to an average coverage of 8x using Sequel IIe machines (Pacific Biosciences). Kinetic data was not generated for these genomes.

### Genome Alignment and TRGT Processing

Genomes were aligned to the GRCh38 reference genome assembly using pbmm2 version 1.7.0. Tandem repeats were genotyped using TRGT version 0.3.3. Tandem repeat loci genotyped were specified by the Adotto catalog version 1.0 (https://zenodo.org/records/7521434) with loci padded by 25bp on each side and loci longer than 1,000bp in the GRCh38 reference genome removed to accommodate a limitation of the version of TRGT used.

### TRGT post-processing to create fuzzy segmentations

TRGT outputs the sequence of each allele for each of the loci specified in the input catalog. We performed k-mer counting to identify all k-mers of length 2 – 12bp present in each allele and retained those who spanned a total of at least 10% of the allele sequence. We then used these ‘major k-mers’ to seed a fuzzy segmentation algorithm. We identified repeating stretches of each motif identified as a ‘major k-mer’ allowing up to one nucleotide of mismatch every four repeat units. These formed our initial segments. Then, we recursively merged the intervals with matching motifs and groomed boundaries between motifs. To determine boundaries between motifs, we gave priority to segments with longer motif lengths and secondarily to segments with longer spans. As an example, a 6-mer followed directly by a 3-mer comprised of the final 3 characters of the 6-mer would finish its segment with a complete unit before the 3-mer segment begins.

### TRGT post-processing to calculate longest pure segment length

TRGT outputs the sequence of each allele for each of the loci specified in the input catalog. We performed k-mer counting to identify all k-mers of length 2 – 12bp present in each allele and retained those who spanned a total of at least 10% of the allele sequence. These were termed ‘major k-mers’. For any alleles where no such ‘major k-mers’ were found, the reference motifs specified in the Adotto catalog were used. The allele sequence output by TRGT was then passed along with the list of ‘major k-mers’ to tr-purity version 1.0^6^. The tr-purity program identifies pure repeat segments and its output was groomed to determine the largest such pure segment for each allele as well as which ‘major k-mer’ composed the segment.

### LPS Motif Simplification

In this schema, motifs are grouped together that are equivalent by any process of reduction, shifting, or reverse complementation and then are written in minimal alphabetical order. To create statistically supported distributions of repeat variations, we also only used the most common LPS motif observed in alleles at each locus (presumed wildtype).

### Constraint model development

The Nucleotide Transformer V2 model with 500 million parameters trained on multi-species data was downloaded from Hugging Face^40^. Using Pytorch Lightning, the model was fine-tuned to simultaneously predict six regression tasks for each input TR locus. These tasks were the values of 1) the standard deviation of the length of the longest pure segment (LPS) for the most common LPS motif; 2) the LPS motif variability; 3) the number of unique motifs observed as the LPS motif; 4) the standard deviation of the length of the total span of the locus; 5) the standard deviation of the LPS across all motifs observed at that locus; and 6) the Composition Polymorphism Score (CPS) defined by Dolzhenko and colleagues as a measure of motif variability. TR loci within 10kb of a gene as defined by Gencode genes basic annotation version 43 were held out. Loci more than 10kb away from a gene were then split into a training dataset composed of 90% of the loci and a validation dataset composed of 10% of the loci. For each locus, the Nucleotide Transformer V2 model was given the tandem repeat sequence as present in the GRCh38 reference genome padded by 75bp on each side. The training and validation sets were further filtered to remove data outliers in the following manner: the values for measures 1, 4 and 5 had to be below the 99^th^ percentile in the training set and the values for measures 2, 3, and 6 had to be below the 99.9^th^ percentile in the training set. Values were then standardized to facilitate prediction (training mean subtracted and then divided by the standard deviation in the training set).

The model was trained for 5 epochs using the AdamW optimizer set with a learning rate of 1e-6. The loss function was the sum of the mean-squared error terms of each of the six regression heads. Performance on the validation set was recorded after each epoch. The trained model was then used on the set of loci within 10kb of genes to predict the “expected” values of variation for each of the six measures, though only measures 5 and 6 are utilized for the manuscript.

## Supplementary Material

Supplement 1

Supplement 2

Supplement 3

Supplement 4

## Data Availability

The data created through the All of Us Program Long Read Data release CDRv7 (April 2023: https://support.researchallofus.org/hc/en-us/articles/14769699298324-v7-Curated-Data-Repository-CDR-Release-Notes-2022Q4R9-versions) are available through the All of Us Research Program researcher workbench (https://researchallofus.org/). Code to reproduce the results presented in this manuscript as well as the resulting data files will be made available as featured workspaces through the All of Us Research Program researcher workbench (https://researchallofus.org/). Additional data that support the findings of this study are available on request from the corresponding authors (M.C.D. and S.Z.).

## References

[1] Vegezzi E. Neurological Disorders Caused by Novel Non-Coding Repeat Expansions: Clinical Features and Differential Diagnosis. Lancet Neurol vol. 23 www.thelancet.com/neurology (2024). 10.1016/S1474-4422(24)00167-4 38876750

[2] Depienne C. & Mandel J. L. 30 years of repeat expansion disorders: What have we learned and what are the remaining challenges? American Journal of Human Genetics vol. 108 764–785 Preprint at 10.1016/j.ajhg.2021.03.011 (2021). 33811808 PMC8205997

[3] Cui Y. A genome-wide spectrum of tandem repeat expansions in 338,963 humans. Cell 187, 2336–2341.e5 (2024). 38582080 10.1016/j.cell.2024.03.004 PMC11065452

[4] Ziaei Jam H. A deep population reference panel of tandem repeat variation. Nat Commun 14, (2023). 10.1038/s41467-023-42278-3 PMC10593948 37872149

[5] Verkerk A. J. M. H. Identification of a Gene (FMR-1) Containing a CGG Repeat Coincident with a Breakpoint Cluster Region Exhibiting Length Variation in Fragile X Syndrome. Cell vol. 65 (1991). 10.1016/0092-8674(91)90397-h 1710175

[6] Dolzhenko E. Characterization and visualization of tandem repeats at genome scale. Nat Biotechnol (2024) doi:10.1038/s41587-023-02057-3. PMC11921810 38168995

[7] English A. C. Analysis and benchmarking of small and large genomic variants across tandem repeats. Nat Biotechnol (2024) doi:10.1038/s41587-024-02225-z. PMC11952744 38671154

[8] Pellerin D. A common flanking variant is associated with enhanced stability of the FGF14-SCA27B repeat locus. Nat Genet 56, 1366–1370 (2024). 38937606 10.1038/s41588-024-01808-5 PMC11440897

[9] Sulovari A. Human-specific tandem repeat expansion and differential gene expression during primate evolution. Proc Natl Acad Sci U S A 116, 23243–23253 (2019). 31659027 10.1073/pnas.1912175116 PMC6859368

[10] Corbett M. A. Intronic ATTTC repeat expansions in STARD7 in familial adult myoclonic epilepsy linked to chromosome 2. Nat Commun 10, (2019). 10.1038/s41467-019-12671-y PMC6820779 31664034

[11] Sato N. Spinocerebellar Ataxia Type 31 Is Associated with ‘Inserted’ Penta-Nucleotide Repeats Containing (TGGAA)n. Am J Hum Genet 85, 544–557 (2009). 19878914 10.1016/j.ajhg.2009.09.019 PMC2775824

[12] Loureiro J. R. Mutational mechanism for DAB1 (ATTTC) n insertion in SCA37: ATTTT repeat lengthening and nucleotide substitution. Hum Mutat 40, 404–412 (2019). 30588707 10.1002/humu.23704

[13] Flanigan K. Autosomal Dominant Spinocerebellar Ataxia with Sensory Axonal Neuropathy (SCA4): Clinical Description and Genetic Localization to Chromosome 16q22.1. Am. J. Hum. Genet 59, 392–399 (1996). 8755926 PMC1914712

[14] Figueroa K. P. A GGC-repeat expansion in ZFHX3 encoding polyglycine causes spinocerebellar ataxia type 4 and impairs autophagy. Nature Genetics vol. 56 1080–1089 Preprint at 10.1038/s41588-024-01719-5 (2024). 38684900

[15] Wallenius J. Exonic trinucleotide repeat expansions in ZFHX3 cause spinocerebellar ataxia type 4: A poly-glycine disease. Am J Hum Genet 111, 82–95 (2024). 38035881 10.1016/j.ajhg.2023.11.008 PMC10806739

[16] Chen Z. Adaptive Long-Read Sequencing Reveals GGC Repeat Expansion in ZFHX3 Associated with Spinocerebellar Ataxia Type 4. Movement Disorders 39, 486–497 (2024). 38197134 10.1002/mds.29704

[17] Paucar M. Spinocerebellar ataxia type 4 is caused by a GGC expansion in the *ZFHX3* gene and is associated with prominent dysautonomia and motor neuron signs. J Intern Med (2024) doi:10.1111/joim.13815. 38973251

[18] Tan D. CAG Repeat Expansion in THAP11 Is Associated with a Novel Spinocerebellar Ataxia. Movement Disorders 38, 1282–1293 (2023). 37148549 10.1002/mds.29412

[19] Stevanin G. The Gene for Spinal Cerebellar Ataxia 3 (SCA3) Is Located in a Region of ‘−3 cM on Chromosome 14q24.3-q32.2. Am.J. Hum. Genet 56, 193–201 (1995). 7825578 PMC1801316

[20] Zu L., Figueroa K. P., Grewal R. & Pulst S.-M. Mapping of a New Autosomal Dominant Spinocerebellar Ataxia to Chromosome 22. Am. J. Hum. Genet 64, 594–599 (1999). 9973298 10.1086/302247 PMC1377770

[21] Serrano-Munuera C. New subtype of spinocerebellar ataxia with altered vertical eye movements mapping to chromosome 1p32. JAMA Neurol 70, 764–771 (2013). 23700170 10.1001/jamaneurol.2013.2311

[22] Hirano R. Fine mapping of 16q-linked autosomal dominant cerebellar ataxia type III in Japanese families. Neurogenetics 5, 215–221 (2004). 15455264 10.1007/s10048-004-0194-z

[23] Pellerin D. Deep Intronic FGF14 GAA Repeat Expansion in Late-Onset Cerebellar Ataxia . New England Journal of Medicine 388, 128–141 (2023). 36516086 10.1056/NEJMoa2207406 PMC10042577

[24] Cortese A. Biallelic expansion of an intronic repeat in RFC1 is a common cause of late-onset ataxia. Nat Genet 51, 649–658 (2019). 30926972 10.1038/s41588-019-0372-4 PMC6709527

[25] Ricker K. Linkage of proximal myotonic myopathy to chromosome 3q. Neurology 52, (1999). 10.1212/wnl.52.1.170 9921867

[26] Sone J. Long-read sequencing identifies GGC repeat expansions in NOTCH2NLC associated with neuronal intranuclear inclusion disease. Nat Genet 51, 1215–1221 (2019). 31332381 10.1038/s41588-019-0459-y

[27] Depienne C. Familial cortical myoclonic tremor with epilepsy The third locus (FCMTE3) maps to 5p. Neurology 74, 2000–2003 (2010). 20548044 10.1212/WNL.0b013e3181e396a8

[28] Guerrini R. Autosomal dominant cortical myoclonus and epilepsy (ADCME) with complex partial and generalized seizures A newly recognized epilepsy syndrome with linkage to chromosome 2p11.1-q12.2. Brain 124, 2459–2475 (2001). 11701600 10.1093/brain/124.12.2459

[29] Mikami M. Localization of a Gene for Benign Adult Familial Myoclonic Epilepsy to Chromosome 8q23.3-q24.1. Am. J. Hum. Genet 65, 745–751 (1999). 10441581 10.1086/302535 PMC1377981

[30] Sirugo G. Mapping the Friedreich ataxia locus (FRDA) by linkage disequilibrium analysis with highly polymorphic microsatellites. Biomed & Pharmacother 48, 219–224 (1994). 10.1016/0753-3322(94)90136-8 7999982

[31] Gusella J. F. A polymorphic DNA marker genetically linked to Huntington’s disease. Nature 306, (1983). 10.1038/306234a0 6316146

[32] Hosler B. A. Linkage of familial amyotrophic lateral sclerosis with frontotemporal dementia to chromosome 9q21-q22. JAMA : the journal of the American Medical Association*.* 284, 1664–1669 (2000). 11015796 10.1001/jama.284.13.1664

[33] Fazal S. RExPRT: a machine learning tool to predict pathogenicity of tandem repeat loci. Genome Biol 25, 39 (2024). 38297326 10.1186/s13059-024-03171-4 PMC10832122

[34] Jensen T. D. Integration of transcriptomics and long-read genomics prioritizes structural variants in rare disease. medRxiv (2024) doi:10.1101/2024.03.22.24304565. PMC12047269 40113264

[35] Fotsing S. F. The impact of short tandem repeat variation on gene expression. Nat Genet 51, 1652–1659 (2019). 31676866 10.1038/s41588-019-0521-9 PMC6917484

[36] Clark R. M., Bhaskar S. S., Miyahara M., Dalgliesh G. L. & Bidichandani S. I. Expansion of GAA trinucleotide repeats in mammals. Genomics 87, 57–67 (2006). 16316739 10.1016/j.ygeno.2005.09.006

[37] Eichler E. E. Length of uninterrupted CGG repeats determines instability in the FMR1 gene. Nat Genet 8, 88–94 (1994). 7987398 10.1038/ng0994-88

[38] Karczewski K. J. The mutational constraint spectrum quantified from variation in 141,456 humans. Nature 581, 434–443 (2020). 32461654 10.1038/s41586-020-2308-7 PMC7334197

[39] Havrilla J. M., Pedersen B. S., Layer R. M. & Quinlan A. R. A map of constrained coding regions in the human genome. Nat Genet 51, 88–95 (2019). 30531870 10.1038/s41588-018-0294-6 PMC6589356

[40] Dalla-Torre H. The Nucleotide Transformer: Building and Evaluating Robust Foundation Models for Human Genomics. bioRxiv (2023) doi:10.1101/2023.01.11.523679. PMC11810778 39609566

