# Supplementary material for: Detailed tandem repeat allele profiling in 1,027 long-read genomes reveals genome-wide patterns of pathogenicity
Source: bioRxiv. 2025 Jan 20:2025.01.06.631535. Preprint. [Version 2] doi: 10.1101/2025.01.06.631535 (PMC11760257; doi:10.1101/2025.01.06.631535)
Supplement: Supplement 4 [file media-4.pdf]

Supplementary Results

Length variation among 3 billion tandem repeat alleles

Most prior work on genome-wide polymorphism of TRs has focused on length variation due to the limitations of short-read sequencing: even algorithms that accurately estimate allele lengths beyond read length are not able to accurately parse complex patterns of motif variation or return consensus sequences for these longer alleles. Long-read sequencing, on the other hand, is not subject to these limitations, allowing us to investigate these complexities in our dataset.

Most TR loci in any individual are below 100bp (Supplementary Figure 1C), but tens of thousands of tandem repeats between 150 and 500bp are still observed in each individual, with further hundreds of thousands of tandem repeat loci that are over 500bp. These loci over 500bp are mostly larger low-complexity regions and regions with numerous neighboring tandem repeats. Algorithms to estimate the length of tandem repeats using short-read genome data become less accurate for tandem repeats longer than the read length – most commonly 150bp. Therefore, our observation of hundreds of thousands of loci beyond this length in any given individual shows that there are many loci not covered or well-estimated in current datasets.

Since short-read data tends to struggle to accurately size TRs over ~175bp, we investigated the frequency of TRs that are shorter than 175bp in >99% of alleles but expand to be greater than 175bp in <1% of alleles. These types of events can be difficult to catch with short-read genome data, but are important for selecting rarely expanded loci that may potentially demonstrate pathogenicity. We observe a median of 150 such rarely-expanded loci per individual (Supplementary Figure 1D), a much higher estimate than our previous findings of three rare TR expansions per individual based on short-read data[1]. Additionally, the saturation plot in Supplementary Figure 1E shows that the set of TRs rarely exceeding that 175bp threshold is continuing to grow at a high rate even after inspecting these 1,027 individuals. Clearly, a much larger cohort of individuals subjected to long-read sequencing will be required to reach saturation of the set of TRs that can expand beyond 175bp.

Motif variation is a major source of polymorphism at tandem repeat loci

The power of analyzing TRs with long-read genome sequencing at scale lies in the unbiased identification of novel motifs within TRs. Using the consensus allele sequences produced by TRGT, we calculated the longest pure segment (LPS) of each allele and the motif of which it was composed. We then compared the observed motifs that produced each LPS and plotted their frequency of divergence from the reference motif (Supplementary Figure 2A). We found that 40.8% of loci had no non-reference motifs that accounted for the LPS in any of their alleles, suggesting motif invariance. In contrast, 26.3% of loci had non-reference LPS motifs in over 90% of their alleles, suggesting either high degrees of motif polymorphism at those loci or misspecification of the reference motifs. Most interesting were the 23.0% of loci where in less than 1% of alleles, a non-reference LPS motif was observed. These loci exemplify the kind of motif polymorphism that would be hard to detect with short-read sequencing technologies and may also demonstrate pathogenicity as observed in diseases such as CANVAS, FAME, SCA31, and SCA37.

We next examined the periodicity of the LPS motifs for each locus. For example, if a locus has a 5bp reference motif, a non-reference LPS motif that is also 5bp long represents a substitution change. This is a distinct motif, but not a novel period. In contrast, a non-reference LPS motif of 6bp represents an insertion, which changes the period (Supplementary Figure 2B). One further example is if a substitution occurs every other motif copy, then we get a 10bp non-reference LPS motif. This we also consider to be of the same period as the reference motif and refer to these scenarios as “interleaved” motif changes. Inspecting the allele database in this way, we found that loci where non-reference LPS motifs were rare were much more likely to harbor period-shifting non-reference LPS motifs than loci where non-reference LPS motifs were ubiquitous (Supplementary Figure 2C-D). This supports the notion that a large percentage of the loci with ubiquitous observation of non-reference LPS motifs are merely poorly annotated – likely with a reference motif that has a substitution every (or every few) motif periods away from the most common motif.

Furthermore, splitting out non-reference motifs into those that are substitutions every occurrence and those that are interleaved substitutions every few occurrences, we see that the interleaved changes make up nearly three-quarters of the non-reference LPS motifs at loci with ubiquitously-altered motifs, while that number is closer to 45% among loci with rare non-reference motifs (Supplementary Figure 3A). The loci with rare non-reference motifs have a much larger share of non-reference LPS motifs that shift the motif by a single base (a 1bp insertion or deletion every motif occurrence).

Subdividing the interleaved non-reference motifs to show how often they occur every other repeat unit, every third repeat unit, and so on, we observe that for both rarely- and ubiquitously-altered motif loci, they tend to decrease the motif size rather than increase it (Supplementary Figure 3B). This suggests that the catalog utilized contains many loci described by overly-complex repeat motifs that are better characterized by a simpler motif that evenly divides the longer motif span (such as a 9bp motif being reduced to a 3bp motif). This highlights the difficult problem of defining a catalog of TRs using only a reference genome or a single individual. We can more robustly determine what are the underlying motifs driving TR variation when using population data. We expect that shorter motifs are likely to better fit the data at a population level.

To determine whether the set of non-reference LPS motifs has been comprehensively observed in these 1,027 individuals, we performed a saturation curve analysis (Supplementary Figure 4A). This indicates that approximately 260 non-reference LPS motifs are still being observed with each additional sample that is added. This suggests that there is still a large array of novel motifs left to be observed that can compose the longest pure segment of their respective TR. Supplementary Figure 4B shows that loci exhibiting five or more non-reference LPS motifs across the population tend to have greater length variance as well.

Variation at known pathogenic TR loci in healthy individuals

Among the set of 62 known pathogenic TRs examined in this study, there existed a wide spectrum of allele lengths within these 1,027 genomes with, predictably, noncoding loci displaying the most variation in length and the largest repeat sizes (Supplementary Figure 8).

Sequence level examinations of known pathogenic loci have garnered profound insights with clinical implications in examples such as in *FMR1* and *HTT* [2,3]. In the *FMR1* 5'UTR CGG repeat locus, AGG interruptions within the CGG repeat offer stabilizing effects during transmission. The risk of premutation alleles expanding to full mutation alleles exponentially increases in alleles lacking AGG interruptions[4]. We have analyzed *FMR1* repeat sequences to exemplify repeat variability at high resolution in a healthy control population. The most common repeat lengths in our cohort correspond well with literature reports (29-31 repeat units, 63.5% of all alleles). There was a range of AGG interruptions from 0 to a maximum of 5 interruptions. The proportion of alleles with 0, 1, 2, 3, 4, or 5 interruptions were 3.7%, 23%, 70%, 2.6%, 0.7%, and 0.1% respectively (Supplementary Figure 9A). However, the frequency of interruptions differed between repeat length groups (Supplementary Figure 9B). Smaller alleles (14-23 units, 7% of alleles) predominantly consisted of 0-1 AGG interruption alleles. Alleles with 24-34 units (84% of alleles) mainly consisted of 1-2 interruptions. There was more variability in the number of AGG interruptions in high normal (35-44, 7% of alleles) and gray zone (45-54, 1.3% of alleles) ranges with 33% and 36% of alleles having 3 or more AGG interruptions respectively. Within the gray zone range, 32% of alleles had four or more AGG interruptions. While the consequences of sequence interruptions at the *FMR1* locus have been largely elucidated, these analyses suggest that similar exploration of sequence level variation at other loci genome-wide may be beneficial. Such analyses, when coupled with phenotype data, may reveal the missing heritability of complex disorders and be a valuable addition to association/modifier studies.

Network analysis of genes with the most and least polymorphic CAG loci

We wondered if the genes containing the coding CAG TRs with the greatest variation in LPS length differed from those containing less variable coding CAG TRs. We found that the genes containing the known pathogenic coding CAG repeats had, on average, more protein-protein interactions than genes containing coding CAG repeats from the lowest percentile of LPS variation (Supplementary Figure 10A). The 15 genes containing the other high variance coding CAGs labelled in Figure 2B also showed enrichment for increased numbers of protein-protein interactions relative to the genes with coding CAG repeats from the lowest percentile of LPS variation (Supplementary Figure 10A), though to a lesser extent than the known pathogenic TRs. We further found that the genes containing the most variable coding CAGs had fewer steps between pairs of them in the protein-protein interaction network than the genes containing the least-variable coding CAGs (Supplementary Figure 10B).

EP400 expansion clinical characteristics

The proband, labeled I.1 in the pedigree (Figure 4A), came from a family with a history of degenerative ataxia in his father and paternal grandfather. He was one of 5 siblings, of which 3 had the disease. He was first clinically assessed at 61 years of age. He noticed in his late 40's that his walking was unsteady, and he started having falls. His speech became dysarthric aged late 50's. He had to retire aged 58 years. At that age he had an ataxic gait, saccadic smooth pursuit of the eyes, cerebellar dysarthria, and a mild intention tremor. A CT scan of the brain showed atrophy of the cerebellum and pons. Four years later, he had progressed to needing a wheeled walker for mobility. He also had urinary frequency. When last seen at the age of 83

years, he was confined to a wheelchair, and he had severe dysarthria and dysphagia. He had hyperreflexia in all four limbs. He died at the age of 84 years following an aspiration pneumonia.

The daughter of the proband, labelled I.3 in the pedigree (Figure 4A), presented with tremor at the age of 40 years. One year later she developed speech difficulty. Shortly after that, her gait became unsteady. Examination showed cerebellar dysarthria, mildly unsteady gait, and brisk deep tendon reflexes in all four limbs. MRI of the brain was unremarkable. One year later, she had progressed only slightly. When she was last seen at the age of 49 years, she had a wide-based unsteady gait, marked dysarthria, saccadic smooth pursuit, and intention tremor bilaterally. She had started using a wheelchair for longer distances.

TR motif constraint

TR motif constraint follows a different pattern than the length constraint. TRs are overwhelmingly constrained for variation against motif changes, with 84% of TRs exhibiting an observed-to-expected ratio below 1 for motif variation and 48% showing no motif polymorphism at all. Supplementary Figure 13A plots this distribution of observed-to-expected scores, but the 48% of the data where observed Composition Polymorphism Score (CPS) was 0 are not plotted for the sake of visual clarity. Inspection of the same set of isolated STR loci used in Figure 3 C,D, we again find that known pathogenic, human ab-initio, and CODIS loci are enriched for the bins of highest observed motif variation (Supplementary Figure 13C). But unlike for length variation, this enhanced variation does not sort into clear patterns of constraint, as for motif constraint we observe the majority of loci in each of the categories to be constrained against motif variation (Supplementary Figure 13D). Repeating this analysis on the complete set of TRs shows consistent patterns (Supplementary Figure 16) compared to the isolated STRs (Supplementary Figure 13).

Short-read approaches achieve high precision but low recall at identifying large TRs

ExpansionHunter

Many studies have investigated TR lengths and variation using large cohorts of short-read data. Many approaches exist to profile TRs in short-read data and we will not attempt a comprehensive comparison of such methods in this work, but instead will just use one commonly used method called ExpansionHunter (EH). Previous work has demonstrated EH to be very accurate at predicting tandem repeats shorter than the read length and it typically gives broadening confidence intervals when attempting to estimate increasingly larger allele sizes beyond the read length[5]. Since we had access to 30x Illumina WGS data on 689 of the 1,027 individuals examined in this studied, we sought to compare EH performance with that of TRGT to determine at which size threshold it becomes less accurate and test whether motif variation exacerbates that effect.

For TR alleles that TRGT measures to be shorter than 150bp, EH makes predictions that are highly consistent with TRGT: achieving an overall Pearson R^2 value of 0.977. However, for alleles that TRGT judges to be longer than 150bp, this relationship is greatly diminished (Supplementary Figure 17A). The Pearson R^2 value drops to only 0.096. Many alleles are genotyped with reasonable accuracy by EH, but the allele lengths EH is willing to predict clearly

plateau around 500bp, while TRGT does not (Supplementary Figure 17A). Dissecting the sizing discrepancies between the tools further, we saw that the vast majority of alleles estimated to be shorter than 150bp by TRGT agreed with their EH estimate within 1bp (Supplementary Figure 17B), but the estimates diverged for longer alleles. For alleles over 250bp, they most often differed by more than 16bp. This gradual shift in the magnitude of disagreement between EH and TRGT as alleles lengthen illustrates the challenges of this work with short-read data and the advantages of using a high quality long-read dataset such as ours. There is a small set of alleles in Supplementary Figure 17A on the upper-left corner of the plot which TRGT calls as being over 500bp, but EH calls as being under 100bp. As best we can tell, these are sequencing errors in the HiFi data which produce long homopolymer stretches which we are not convinced are real. Further investigation of such events is necessary.

For the TRGT calls over 150bp, we also plotted the absolute difference between the TRGT allele length and the corresponding EH allele length stratified by TR motif (Supplementary Figure 17C). This view shows that the AAAAT motifs represent some of the most dramatically mis-genotyped loci by EH. We also observe numerous genotyping discrepancies at known pathogenic loci that constitute shifts of several repeat units, which could prove problematic in medical genetics work using EH as a screening tool.

ExpansionHunter Denovo

In large cohorts sequenced by short-read approaches, the ExpansionHunter Denovo (EHDn) tool is often used to identify TRs that are expanded in one or several individuals in pursuit of novel pathogenic repeat expansions[6]. Hence, we investigated the accuracy of identifying large repeats using EHDn. We applied EHDn to 689 short-read genomes, and TRGT to their corresponding long-read counterparts. For this analysis, we calculated precision and recall values at different comparison thresholds.

EHDn does not estimate repeat size, but instead provides a measure of the number of anchored in-repeat-reads (IRRs) detected at a particular locus. High anchored IRR counts at TRs indicate the repeat is larger than the read length (>175 bp). Using TRGT genotypes as the 'truth set', we calculated precision and recall values for different thresholds of EHDn anchored IRRs and TRGT repeat sizes. We found that a measure of five anchored IRRs produced a median precision of 87.83% and recall of 1.02% for identifying repeats of 125 bp or larger among all samples. As we increased the size threshold in increments of 25 bp up to 225 bp, the median precision decreased to 66.41% and the recall increased to 2.88% (Supplementary Figure 18A, C). We also calculated precision and recall values for different anchored IRR thresholds, to measure their ability to identify repeats greater than 175 bp. We found that 5 anchored IRRs had a median precision of 80.95% and recall of 1.94%, and the precision increased with an increase in anchored IRRs, but the recall decreased (Supplementary Figure 18B, D).

While exploring the EHDn TRs, we found a subset that were not genotyped by TRGT that we have labelled as *de novo* TRs. We found that *de novo* TRs comprised 29.94 – 52.88% of all EHDn-identified TRs when filtering for those with anchored IRRs above five. As we increased the threshold to 15 anchored IRRs, the upper range of *de novo* TRs increases to 66.67%, with a median of 45.45% (Supplementary Figure 18E).

Next, we combined all the TRs with five or more anchored IRRs from each sample and removed duplicate loci within each group. Supplementary Figure 19A shows the distribution of TRs that were expanded, not expanded, had a motif mismatch, or were de novo. We then compared all

the cataloged TRs to the *de novo* TRs. We found that *de novo* TRs were more likely to be defined as satellites (odds ratio [OR] = 17.09; 95% confidence interval [CI] = [5.92, 49.33]), and less likely to be defined as simple repeats (OR = 0.5; 95% CI = [0.43, 0.59]) or SINEs (OR = 0.70; 95% CI = [0.61, 0.79]) according to the UCSC repeat database. The majority of these *de novo* TRs were not categorized in this database (Supplementary Figures 19B, C). Upon investigating motif patterns, we found that catalogued TRs were enriched in AAAG (OR = 6.19; 95% CI = [4.39, 8.72]) and AAGGAG (OR = 5.02; 95% CI = [2.33, 10.80]) motifs while *de novo* TRs were enriched in AATGG (OR = 42.41; 95% CI = [18.67, 96.34]) and AACCCT (OR = 4.79; 95% CI = [2.15, 10.67]) motifs (Supplementary Figure 19, D, E). We also discovered that *de novo* TRs were enriched in A+G+T combinations (OR = 5.13; 95% CI = [3.76, 7.00]) in their motifs, as well as A+C combinations (OR = 1.64; 95% CI = [1.39, 2.39]) (Supplementary Figure 20A, B).

We also drew comparisons between TRs that were recalled and those that were not. To do this, we combined all the TRGT genotyped TRs for each sample and categorized them into those that were recalled by EHDn, and those that were not. We found that unrecalled TRs were most enriched in A+T (OR = 11.76; 95% CI = [11.30, 12.25]) or A+C (OR = 15.12; 95% CI = [15.12, 16.54]) nucleotide combinations (Supplementary Figure 20C-D). Upon comparing the GC content of TRs that were recalled and unrecalled, we found surprisingly that recalled TRs have a higher GC percentage in their motifs (Cohen's d = 0.62), are less pure in their motifs (d = -0.59) and are larger in LPS size (d = 3.25) (Supplementary Figure 21A-C). We did not see any significant difference in the number of unique motifs observed at specific loci between the two groups (Supplementary Figure 21D).

Supplementary Figure Legends:

<u>Supplementary Figure 1: Length variation in 3 billion tandem repeat alleles</u>

A) Histogram of median absolute deviation of repeat length for each of the 1.7 million analyzed TR loci. B) Boxplots of median absolute deviation of repeat length for the entire catalog, the known pathogenic loci, the set of loci identified by Sulovari and colleagues to be expanded specifically in humans (human Ab-initio loci), and CODIS loci. C) Bar plot of the mean number of TR alleles of various lengths present in each individual in the cohort. Error bars represent standard deviations. D) Violin plot of the count of loci per individual that are longer than 175bp in that person but are shorter than 175bp in >99% of the cohort. E) Cumulative number of loci identified that exceed 175bp in length in fewer than 1% of individuals in the cohort as individuals are added to the cohort.

<u>Supplementary Figure 2: Motif variation is a major source of polymorphism at tandem repeat loci</u>

A) Variable-width bar plot of the proportion of loci found to harbor motifs not present in the reference catalog. To be counted, a motif must create the longest pure segment observed in an allele. B) Diagram of motif changes to a reference motif that depict it changing into various non-reference motifs which can be of the same period (first example), an interleaved pattern with a period that is a direct multiple of the reference motif (second example), and a non-reference motif period (third example). C and D) Pie charts of the occurrence rate of period changes when

the LPS is a novel motif among loci with C) rarely-altered (<1% of alleles non-reference motif) motifs and D) ubiquitously-altered (>99% of alleles non-reference motif) motifs.

Supplementary Figure 3: Motif variations that alter the period length

A) Grouped bar plot of the occurrence rate of periodicity changes among novel LPS motifs for rarely altered loci (<1% of alleles non-reference motif) and ubiquitously altered loci (>99% of alleles non-reference motif). B) For the substitution and interleaved motif changes only, grouped bar plot of the occurrence rate of various length modifications for rarely altered loci and ubiquitously altered loci.

Supplementary Figure 4: Many novel motifs still remain to be discovered

A) Cumulative number of novel LPS motifs observed as individuals are added to the cohort. B) Boxplot of variation in the length of the LPS among groups of loci which only exhibit the reference motifs, 1-4 novel LPS motifs, 5-9 novel LPS motifs, and 10 or more novel LPS motifs.

Supplementary Figure 5: LPS length variance per motif class for motifs of length 2, 4, and 5bp

A-C) Stripplots of the standard deviation of the length of the LPS for each of the simplified motif classes. Each dot represents one TR locus. For each locus, only the most commonly observed LPS motif is used. A) 2bp motifs. B) 4bp motifs. C) 5bp motifs.

Supplementary Figure 6: LPS length variance per motif class for 6bp motifs

Stripplot of the standard deviation of the length of the LPS for each of the 6bp simplified motif classes. Each dot represents one TR locus. For each locus, only the most commonly observed LPS motif is used.

Supplementary Figure 7: Pathogenic tandem repeats are among the most variable in the genome on a per-motif basis

Scatter plots of the median vs 99th percentile pure repeat lengths for nine different motifs. Disease-associated loci are shown in orange and catalogued loci are shown in green. Several top candidate loci are labelled with their gene name. in each plot. The x=y line is shown as a dashed grey line in each panel.

Supplementary Figure 8: Characterization of variation observed at known pathogenic tandem repeats

Boxplots of repeat length (base pairs) of known pathogenic loci in 2054 chromosomes.

Supplementary Figure 9: Length variation and AGG interruptions in *FMR1*

A) Histogram depicting the frequency of repeat lengths and AGG interruptions in the *FMR1* repeat locus, n=1,675. B) Stacked bar plot showing the proportion of different amounts of AGG interruption in *FMR1* alleles for five different ranges of total repeat length.

Supplementary Figure 10: Network analysis of genes with the most and least polymorphic CAG loci

A) Cumulative frequency plot of the number of protein-protein interactions for the genes containing either the known pathogenic coding CAG TRs (orange), the other high variance coding CAGs identified in Figure 2E (blue), or a set of low variance coding CAGs. B) Distribution

of shortest path lengths between members of the gene sets in panel A across the protein-protein interaction network.

Supplementary Figure 11: Expression variation of *EP400* and *ATXN2*

A) Gene expression levels of *EP400* across many tissues as reported by GTEx. B) Gene expression levels of *ATXN2* across many tissues as reported by GTEx.

Supplementary Figure 12: Characteristics of TR length constraint

A) Histogram of observed length variation (measured by standard deviation of the LPS length, regardless of motif) for loci within 10kb of a gene. B) Histogram of observed-to-expected ratio for length variation for loci within 10kb of a gene. This plot only shows values below 3 for visual clarity. Red vertical lines mark values of 0.333 (representing the threshold of negative selection) and 2.0 (representing the threshold of positive selection). The green vertical line marks the value of 1.0. C) Scatterplot of the observed vs expected length variation for the 16 CODIS loci within 10kb of a gene. The square of the Pearson correlation coefficient for this set of values is 0.85.

Supplementary Figure 13: Characteristics of TR motif constraint

A) Histogram of the observed-to-expected ratio for motif variation for loci within 10kb of a gene. This plot only shows values below 3 for visual clarity. Red vertical lines mark values of 0.333 (representing the threshold of negative selection) and 2.0 (representing the threshold of positive selection). The green vertical line marks the value of 1.0. B) Scatterplot of the observed vs expected motif variation for the 16 CODIS loci within 10kb of a gene. The square of the Pearson correlation coefficient for this set of values is 0.62. C) Grouped bar plot of the percentage of isolated STRs belonging to each of the five specified sets which fall into each of the deciles of observed motif variation. Observed motif variation for each TR is the composition polymorphism score (see methods). D) Grouped bar plot of the percentage of isolated STRs belonging to each of the five specified sets which fall into each of the bins of motif constraint. The observed-to-expected ratio of motif variation for each of the bins are defined as: Constrained (0-0.33), Slightly Constrained (0.33-0.75), Neutral (0.75-1.25), Slightly Extra Variance (1.25-2.0), Extra Variance (over 2.0).

Supplementary Figure 14: TR length constraint results on complete set of genic loci

A) Grouped bar plot of the percentage of TRs belonging to each of the five specified sets which fall into each of the deciles of observed length variation. Observed length variation for each TR is the standard deviation of the length of the longest pure segment. B) Grouped bar plot of the percentage of TRs belonging to each of the five specified sets which fall into each of the bins of length constraint. The observed-to-expected ratio of length variation for each of the bins are defined as: Constrained (0-0.33), Slightly Constrained (0.33-0.75), Neutral (0.75-1.25), Slightly Extra Variance (1.25-2.0), Extra Variance (over 2.0).

Supplementary Figure 15: RExPRT predictions on TR constraint groups

Odds ratio plot of RExPRT scores for TRs identified in the ‘constrained’, ‘neutral’, and ‘extra variance’ bins in Supplementary Figure 14B relative to the complete catalog. The odds ratio value of 1.0 (designated with a dashed line) represents non-significance and any confidence intervals that do not cross that line denote significant results.

Supplementary Figure 16: TR motif constraint results on complete set of genic loci

A) Grouped bar plot of the percentage of TRs belonging to each of the five specified sets which fall into each of the deciles of observed motif variation. Observed motif variation for each TR is the composition polymorphism score (see methods). B) Grouped bar plot of the percentage of TRs belonging to each of the five specified sets which fall into each of the bins of motif constraint. The observed-to-expected ratio of motif variation for each of the bins are defined as: Constrained (0-0.33), Slightly Constrained (0.33-0.75), Neutral (0.75-1.25), Slightly Extra Variance (1.25-2.0), Extra Variance (over 2.0).

Supplementary Figure 17: Short-read vs long-read comparison using ExpansionHunter

A) Comparison of estimated length of tandem repeats from paired short-read and long-read datasets for alleles estimated by TRGT to be above 150bp. Short-read data TR lengths estimated with ExpansionHunter (EH) and long-read data TR lengths estimated with TRGT. B) Grouped bar plot of the proportion of alleles in each category of difference in length between TRGT and EH estimates, stratified by TRGT estimate of allele length. C) Stripplot of difference in estimate of allele length by TRGT and EH for alleles estimated by TRGT to be above 150bp, stratified by motif.

Supplementary Figure 18: Short-read approaches achieve high precision but low recall at identifying large TRs

A-B) Precision of ExpansionHunter Denovo (EHDn) in identifying large TRs. A) Precision of EHDn over a range of threshold TR sizes as determined by TRGT on the long-read data. B) Precision of EHDn at the 175bp threshold size over a range of thresholds of anchored in-repeat-reads. C-D) Recall of EHDn in identifying large TRs. C) Recall of EHDn over a range of threshold TR sizes as determined by TRGT on the long-read data. D) Recall of EHDn at the 175bp threshold size over a range of thresholds of anchored in-repeat-reads. E) Percentage of EHDn calls that did not correspond to any locus genotyped by TRGT (de-novo TRs) over a range of thresholds of anchored in-repeat-reads.

Supplementary Figure 19: Enrichment of repeat classes and repeat motifs in de novo TRs

A) Number of TRs called by EHDn categorized into groups: truly expanded as evidenced by the TRGT call (expanded), not expanded as evidenced by the TRGT call (not expanded), having an alternative motif than that called by TRGT (motif mismatch), or not genotyped by TRGT at all (de novo). B) Enrichment of different repeat classes in the cataloged and de novo TR groups. C) Frequencies of different repeat classes in the cataloged and de novo TR groups. D) Frequencies of significantly observed motifs between the cataloged and de novo TR groups. E) Odds ratios observed for the significantly different motifs between the cataloged and de novo TR groups.

Supplementary Figure 20: Nucleotide compositions of the motifs called by EHDn

A) Frequencies of different nucleotide compositions of motifs in the cataloged and de novo TR groups. B) Odds ratios of the different nucleotide compositions of motifs in the cataloged and de novo TR groups. C) Odds ratios of the different nucleotide compositions of motifs in the recalled and unrecalled TR groups. D) Frequencies of the different nucleotide compositions of motifs in the recalled and unrecalled TR groups.

Supplementary Figure 21: Sequence characteristics of large TRs called and not called by EHDn

A-D) Violin plots of sequence characteristics of TRs determined by TRGT to be longer than 175bp which were identified by (recalled) and not identified by (unrecalled) EHDn: A) GC percentage, B) TR purity, C) length of the longest pure segment, and D) number of unique motifs.

Supplementary Table Legends:

Supplementary Table 1: Total allele lengths at known pathogenic loci.

Lists the distribution of raw allele lengths (in bp) observed for 61 known pathogenic loci across the cohort. For each locus, the ID of the TR is given, along with the name of the corresponding gene, then the allele length in the cohort for the following percentiles is given: 0, 1, 5, 10, 15, 20, 25, 30, 35, 40, 45, 50, 55, 60, 65, 70, 75, 80, 85, 90, 95, 99, 99.9, and 100. The lengths listed refer to the measurements of the genomic region specified by the TRID, which is often not identical to the typical coordinates used for these pathogenic loci. Most loci have values 50bp larger than the repetitive length because of the padding by 25bp on each side of the loci. Some loci contain multiple repetitive regions (e.g., *FMR1* and *HTT*).

Supplementary Table 2: Longest pure segment lengths at known pathogenic loci.

Lists the distribution of longest pure segment lengths (in bp) observed for 61 known pathogenic loci across the cohort. For each locus, the ID of the TR is given (TRID), along with the name of the corresponding gene (TRName), the motif being measured (longestPureSegmentMotif), and the number of alleles in which that motif accounted for the longest pure segment at this locus (N_motif). Each locus can appear numerous times with different motifs accounting for the longest pure segment and the sum of the values of 'N_motif' can reach as high as 2,054 (two alleles for each of 1,027 individuals), though there are missing genotype calls for many loci, so the values do not always sum to 2,054 exactly. For each row, the length of the longest pure segment (for the corresponding motif) in the cohort for the following percentiles is given: 0, 1, 5, 10, 15, 20, 25, 30, 35, 40, 45, 50, 55, 60, 65, 70, 75, 80, 85, 90, 95, 99, 99.9, and 100.

Supplementary Table 3: TR Constraint results for loci within 10kb of genes.

For each of the 923,089 catalog loci within 10kb of a gene, this table lists the observed and expected (predicted) values for standard deviation of LPS length (combined across all LPS motifs) and Composition Polymorphism Score (CPS). It then gives the computed observed-to-expected ratios for these measures of length and motif variation.

Supplementary methods:

ExpansionHunter Denovo and TRGT comparison

We genotyped TRs in 689 matched pairs of samples with both short-read and long-read sequencing. These 689 samples were a subset of the total set of 1,027 samples. TRs in short-read genomes were genotyped with ExpansionHunter (EH) and ExpansionHunter Denovo (EHDn) and TRs in long-read genomes were genotyped with TRGT. We compared repeat sizes

of TRs genotyped by the two different methods, and using TRGT as the ‘truth set’, we calculated precision and recall values for EHDn calls.

For each sample, we filtered EHDn results to select for TRs with at least five anchored in-repeat-reads (IRRs). Next, we used bedtools intersect to find the intersection of any TRs identified by EHDn and TRGT, as well as those that were unique to each tool. For intersecting TRs, we compared motifs as well as reads/sizes produced by each tool. We categorized the EHDn TRs into four groups:

A. Expanded: EHDn TRs that are above the base pair size threshold according to the TRGT calls.

B. Not expanded: EHDn TRs that are below the base pair size threshold according to the TRGT calls.

C. Motif mismatch: EHDn TRs that have a different motif from the corresponding TRGT calls.

D. De novo: EHDn TRs that are not found in the adotto catalog used for the TRGT calls.

The base pair thresholds we used for comparison were 125, 150, 175, 200, and 225 bp. To calculate the sizes using the TRGT data, we counted the lengths of segments of major motifs, allowing for ‘fuzzy matching’. To check if a TR crossed the threshold, we added together the sizes of all segments in that region matching the motif of interest. Using the specified categorizations above, we calculated the precision and recall rates of EHDn at these different thresholds. These metrics were defined as:

1. $$\boldsymbol{Precision = \frac{Expanded\ TRs}{Expanded\ TRs + Not\ expanded\ TRs + Motif\ mismatched\ TRs + De\ novo\ TRs}}$$

2. $$\boldsymbol{Recall = \frac{Expanded\ TRs}{TRGT\ TRs \geq bp\ threshold}}$$

Note that when calculating recall, we only used the larger allele for TRGT data since this dataset is allele specific while EHDn only provides a single read count per TR locus and does not contain information on zygosity. We also repeated this analysis using a threshold of 175 bp, and filtering for EHDn TRs with at least 10 and 15 anchored IRRs.

Comparison between de novo and cataloged TRs

We combined all the TRs above five anchored IRRs for all samples and separated them into de novo TRs, and all other TRs (cataloged). Next, we used bedtools merge to combine any overlapping TRs within groups. To investigate which types of repeats are represented in the different groups, we downloaded the Simple Repeats track from UCSC’s Table Browser. This dataset categorizes repeats by coordinates into different groups. After calculating frequencies of TRs in the different categories, we computed fisher’s exact test odds ratios for each category, comparing the two broad groups (de novo and cataloged).

We also calculated frequencies of different motifs within the two groups and their corresponding odds ratios. The graphs represent only significantly enriched motifs within any of the groups.

Finally we investigated the nucleotide compositions of the motifs and calculated the frequencies and odds ratios in both groups.

Comparison between recalled and unrecalled TRs

We used bedtools intersect to compare EHDn and TRGT TRs for each sample. The resulting files were concatenated then split into recalled and unrecalled groups. Recalled TRs were defined as any EHDn TRs that intersected with TRGT TRs. Bedtools merge was used to combine any overlapping TRs within groups. We investigated nucleotide compositions of the motifs and calculated their frequencies and odds ratios.

To calculate the GC percentage, we took the complete TRGT sequence for each allele, removed the 25bp flanking sequence on either end and counted the number of G and C nucleotides in the sequence. We then divided this by the sequence length, and this gave us the GC content of the allele. We compared the GC content of alleles in the two groups and performed a Wilcoxon test to assess significance and calculated a Cohen's d effect size.

To calculate purity, we again removed the flanking 25bp sequence on either size of the repeat from each allele. We then identified the most common motif within the allele by taking the motif which occurred most frequently. We then counted the number of times the motif occurred and divided it by the length of the sequence and multiplied by 100 to get the percentage purity.

The longest pure segment size was calculated by taking the major motif segments from previous processing and identifying the longest segment of these and calculating its size. These segments allow for some fuzzy matching, or minor interruptions. Finally, the number of unique motifs was calculated by counting the number of motifs per allele. This was counted from the major motifs identified, which was part of previous data preprocessing.

Supplementary References:

1. Fazal, S. et al. Large scale in silico characterization of repeat expansion variation in human genomes. Sci Data 7, 1–14 (2020).

2. Wright, G. E. B. et al. Length of Uninterrupted CAG, Independent of Polyglutamine Size, Results in Increased Somatic Instability, Hastening Onset of Huntington Disease. Am J Hum Genet 104, 1116–1126 (2019).

3. Yrigollen, C. M. et al. AGG interruptions within the maternal FMR1 gene reduce the risk of offspring with fragile X syndrome. Genetics in Medicine 14, 729–736 (2012).

4. Eichler, E. E. et al. Length of uninterrupted CGG repeats determines instability in the FMR1 gene. Nat Genet 8, 88–94 (1994).

5. Dolzhenko, E. et al. ExpansionHunter: A sequence-graph-based tool to analyze variation in short tandem repeat regions. Bioinformatics 35, 4754–4756 (2019).

6. Dolzhenko, E. et al. ExpansionHunter Denovo: A computational method for locating known and novel repeat expansions in short-read sequencing data. Genome Biol 21, 1–14 (2020).

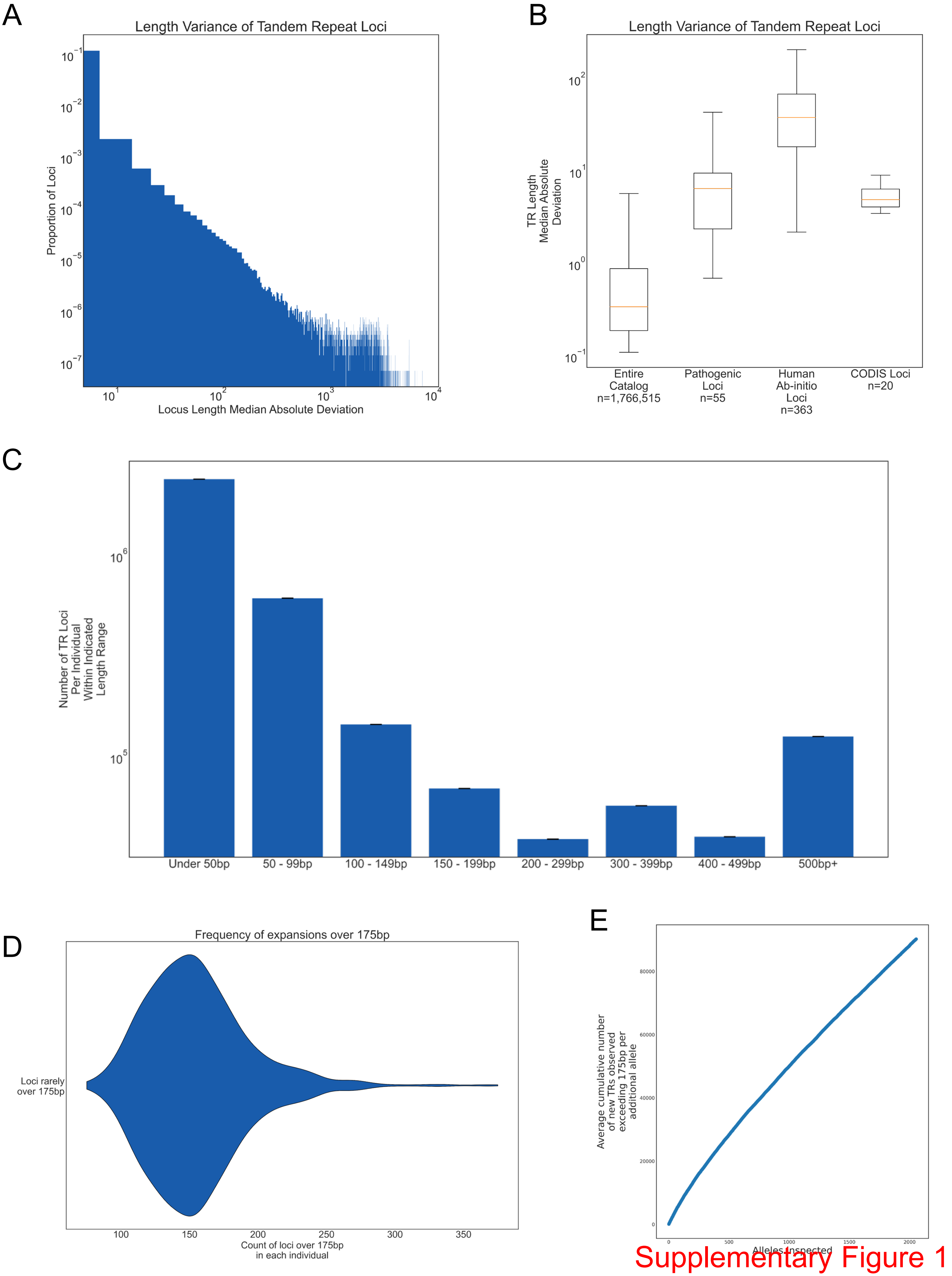

Supplementary Figure 1

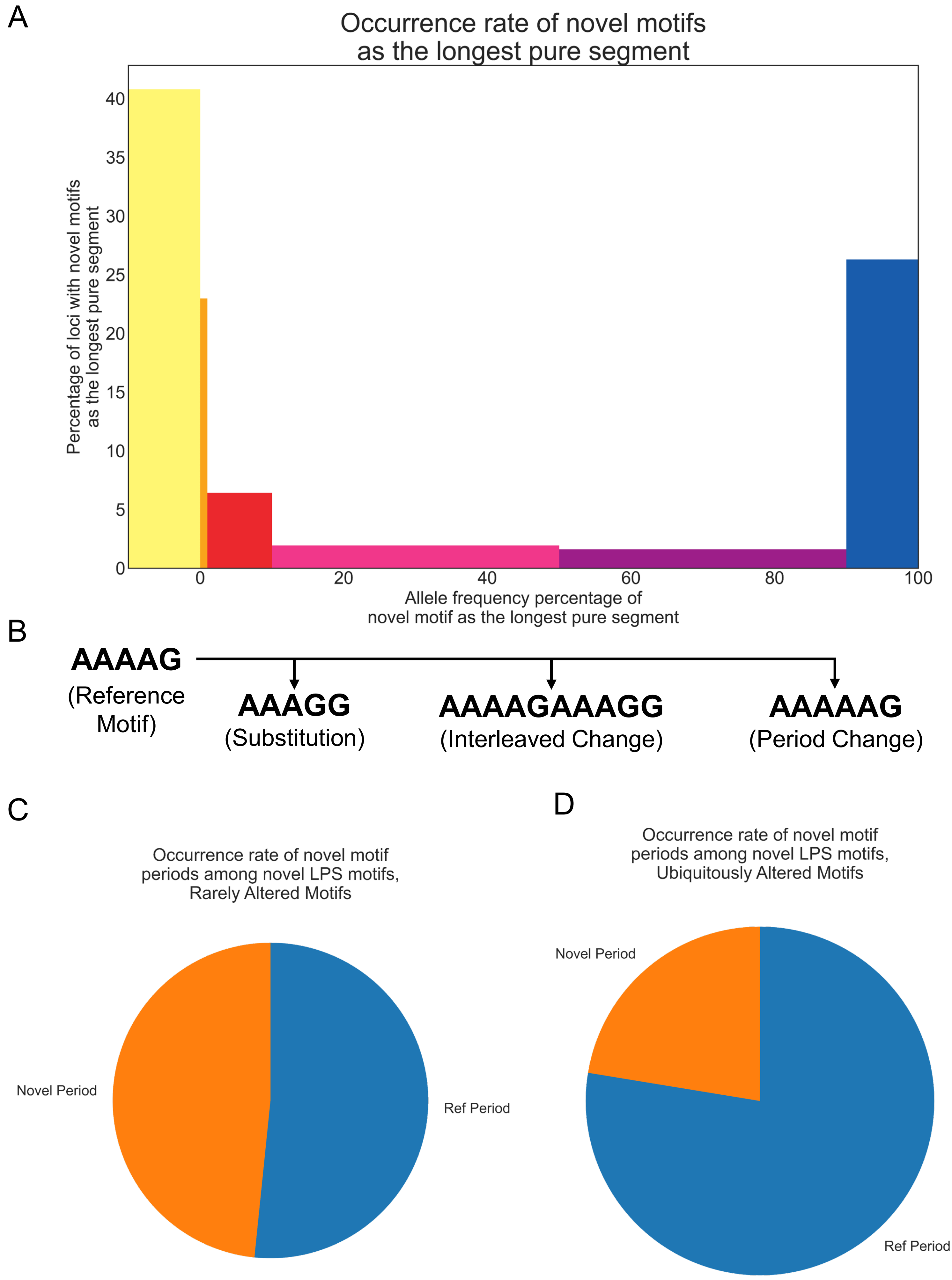

Supplementary Figure 2

A

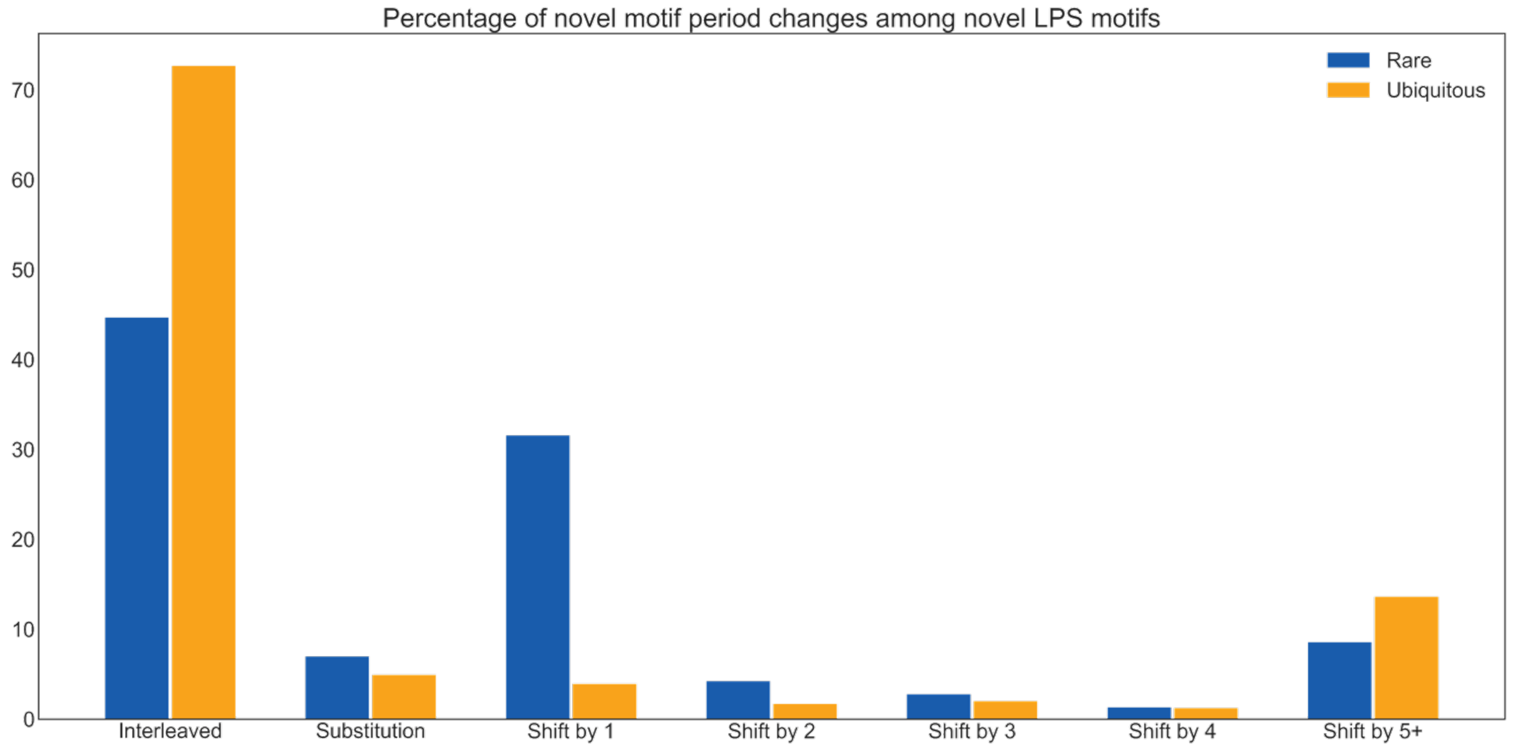

B

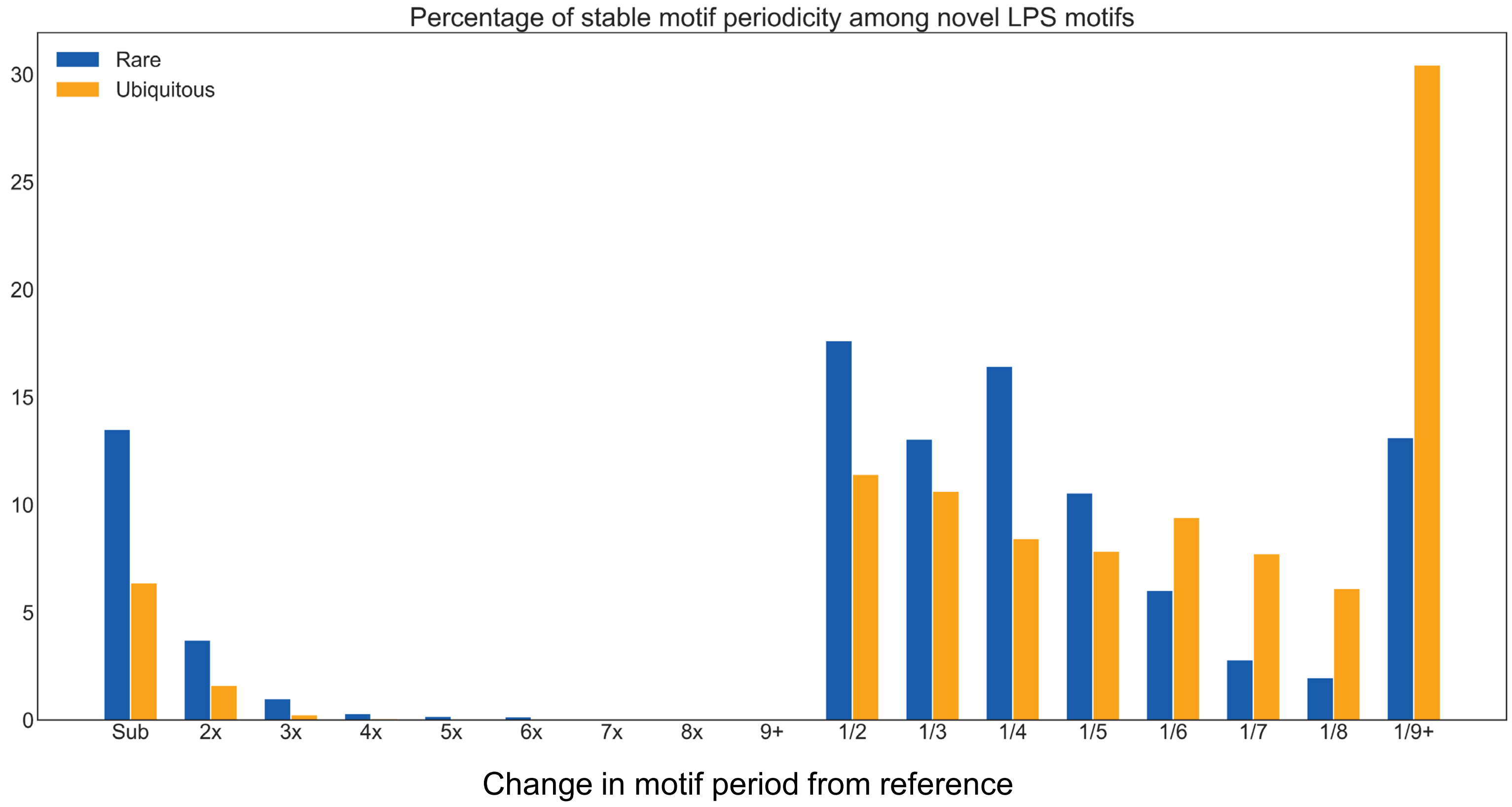

Supplementary Figure 3

A

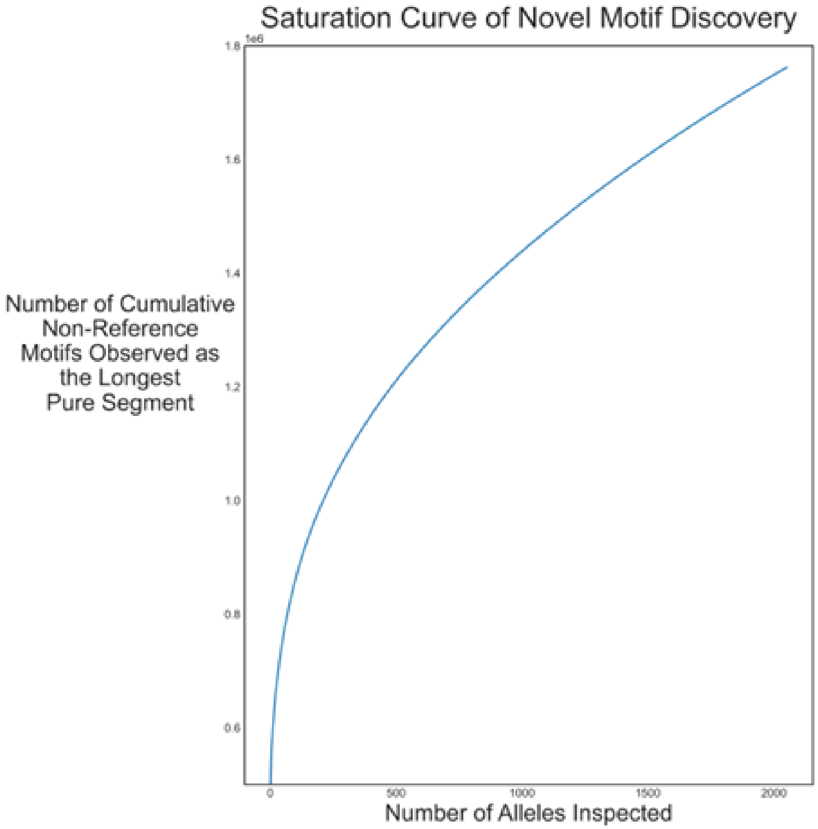

B

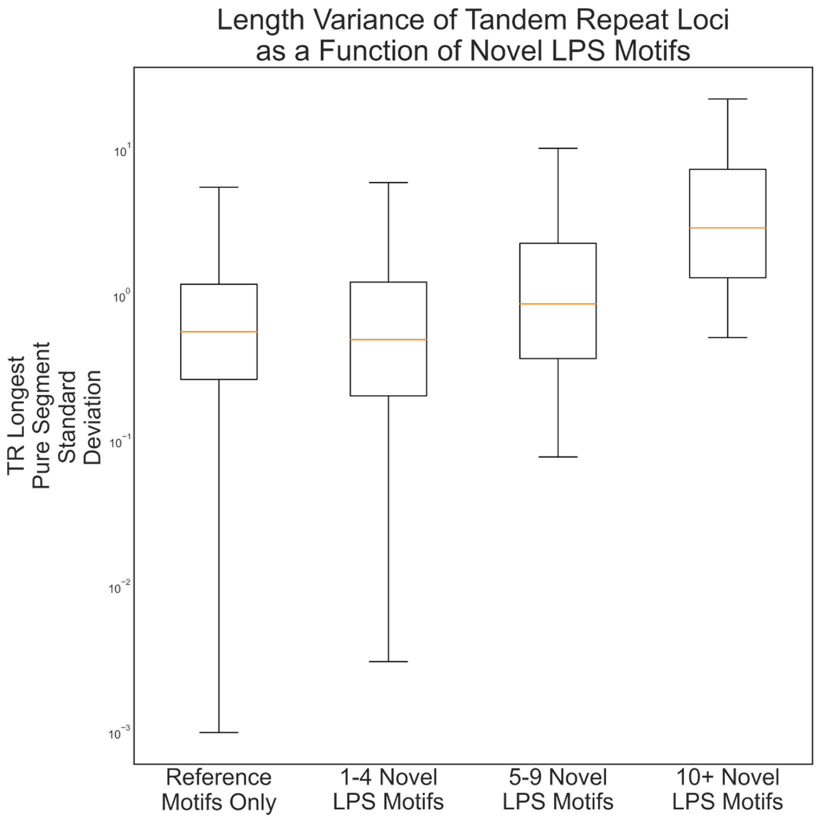

Supplementary Figure 4

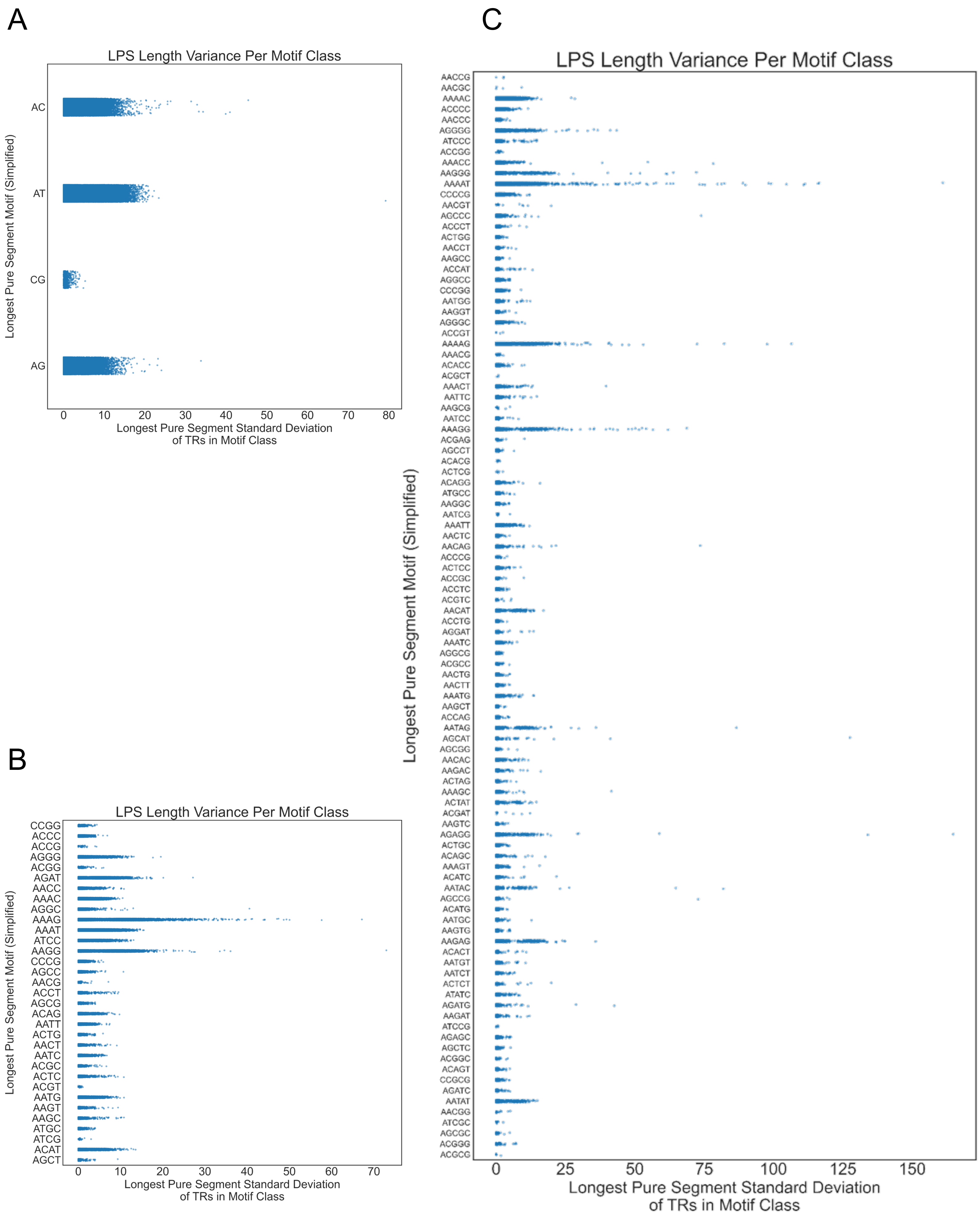

Supplementary Figure 5

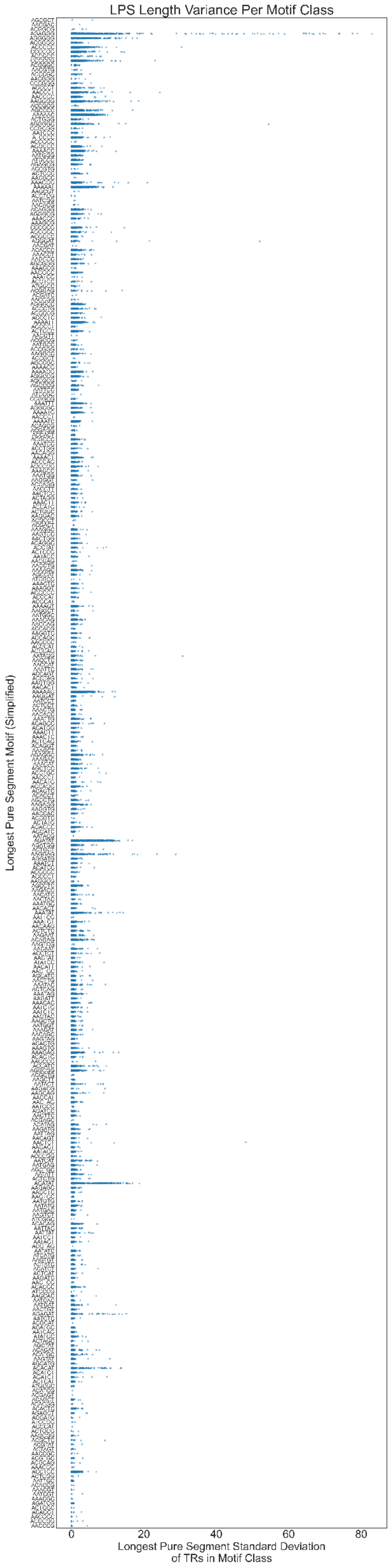

Supplementary Figure 6

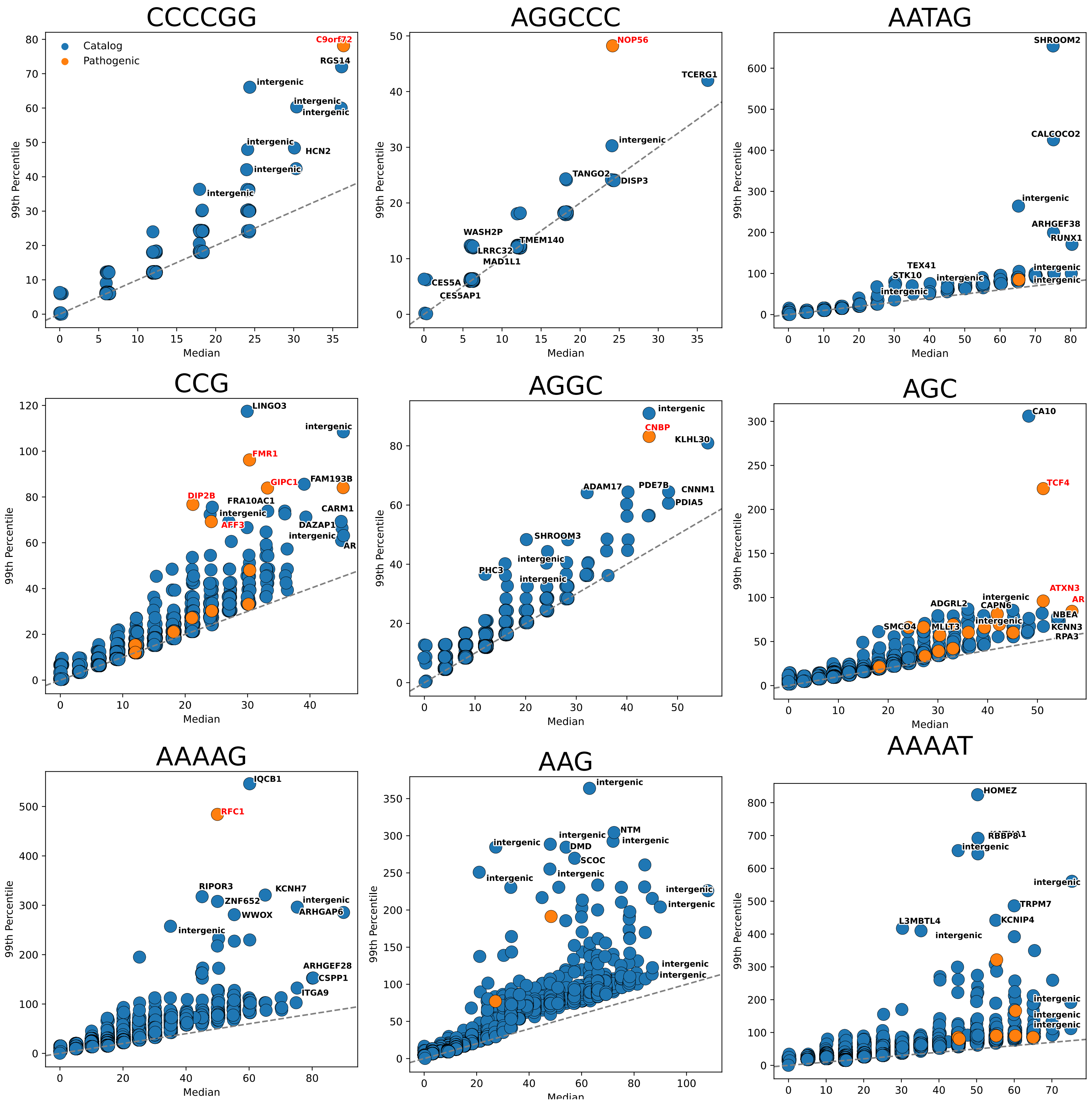

Supplementary Figure 7

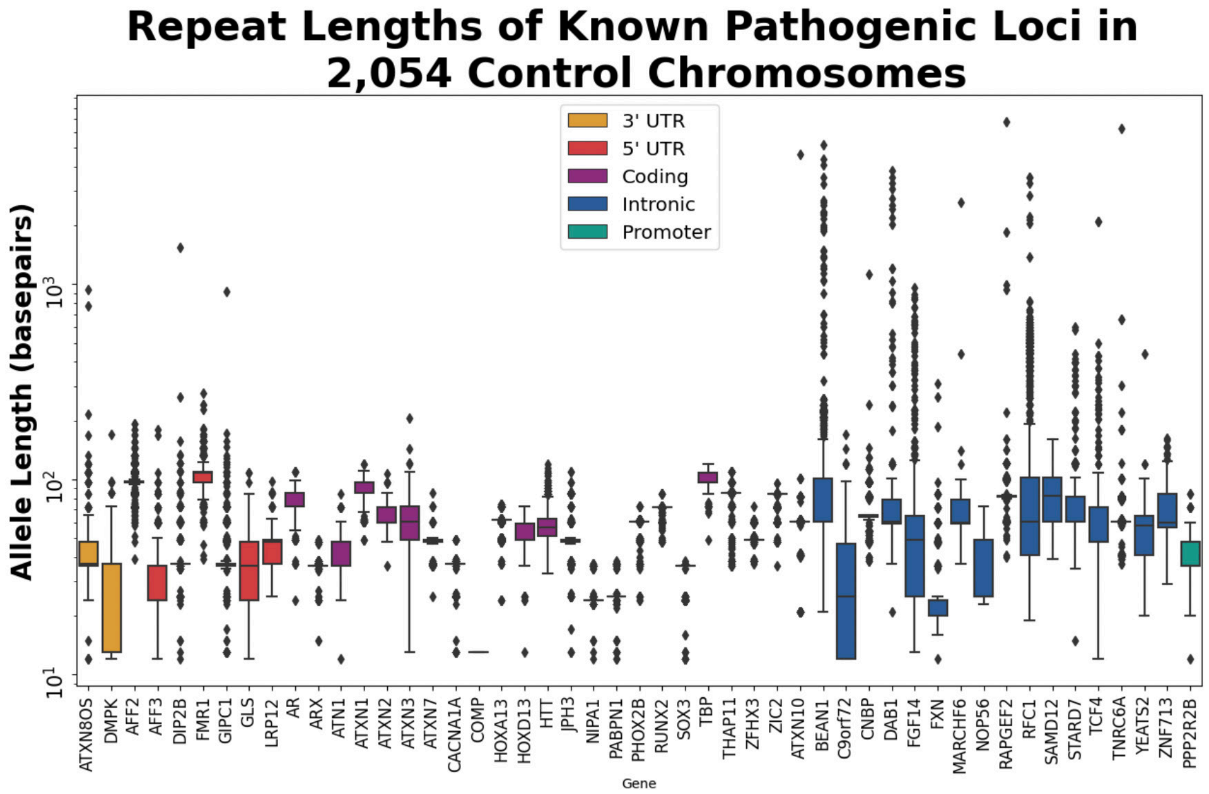

Supplementary Figure 8

A

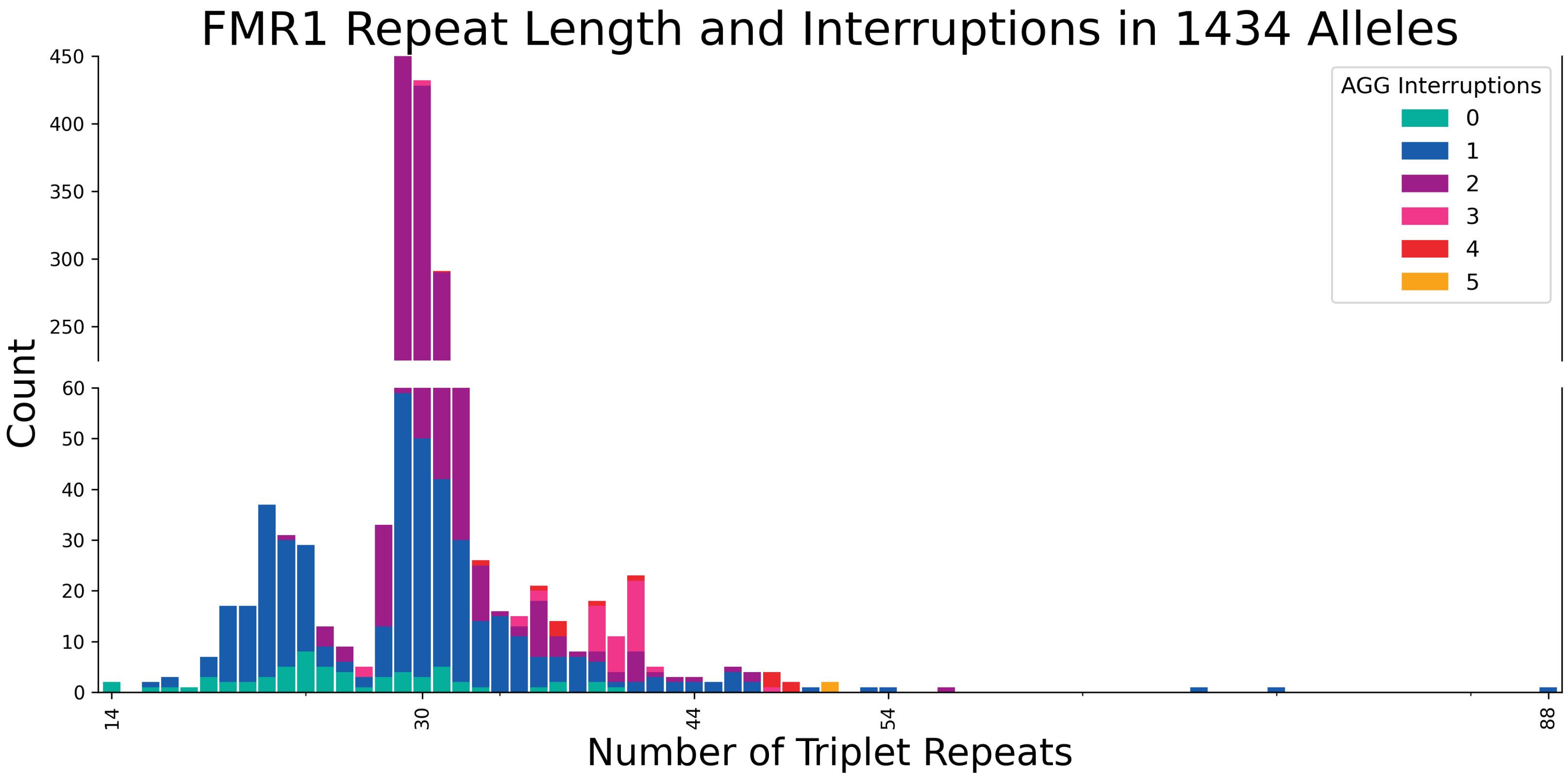

B

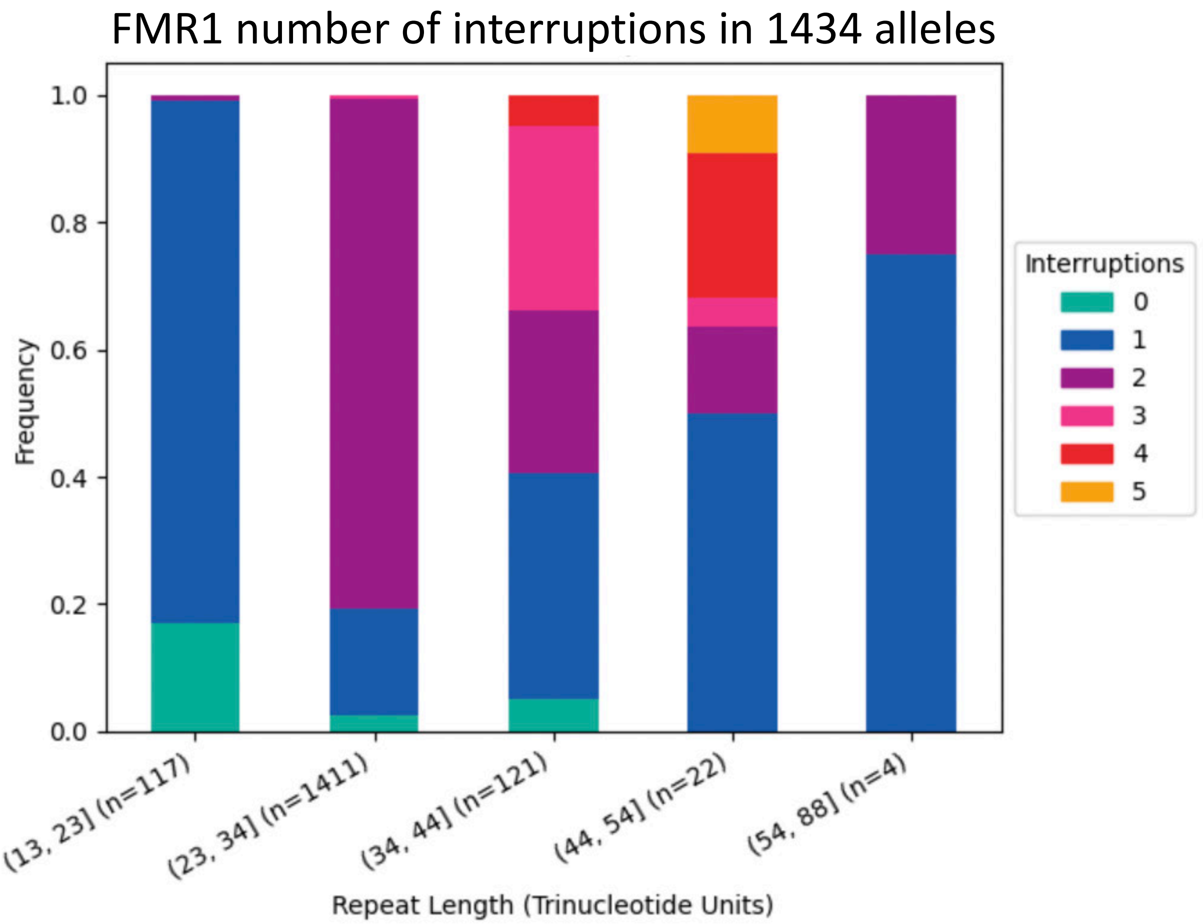

Supplementary Figure 9

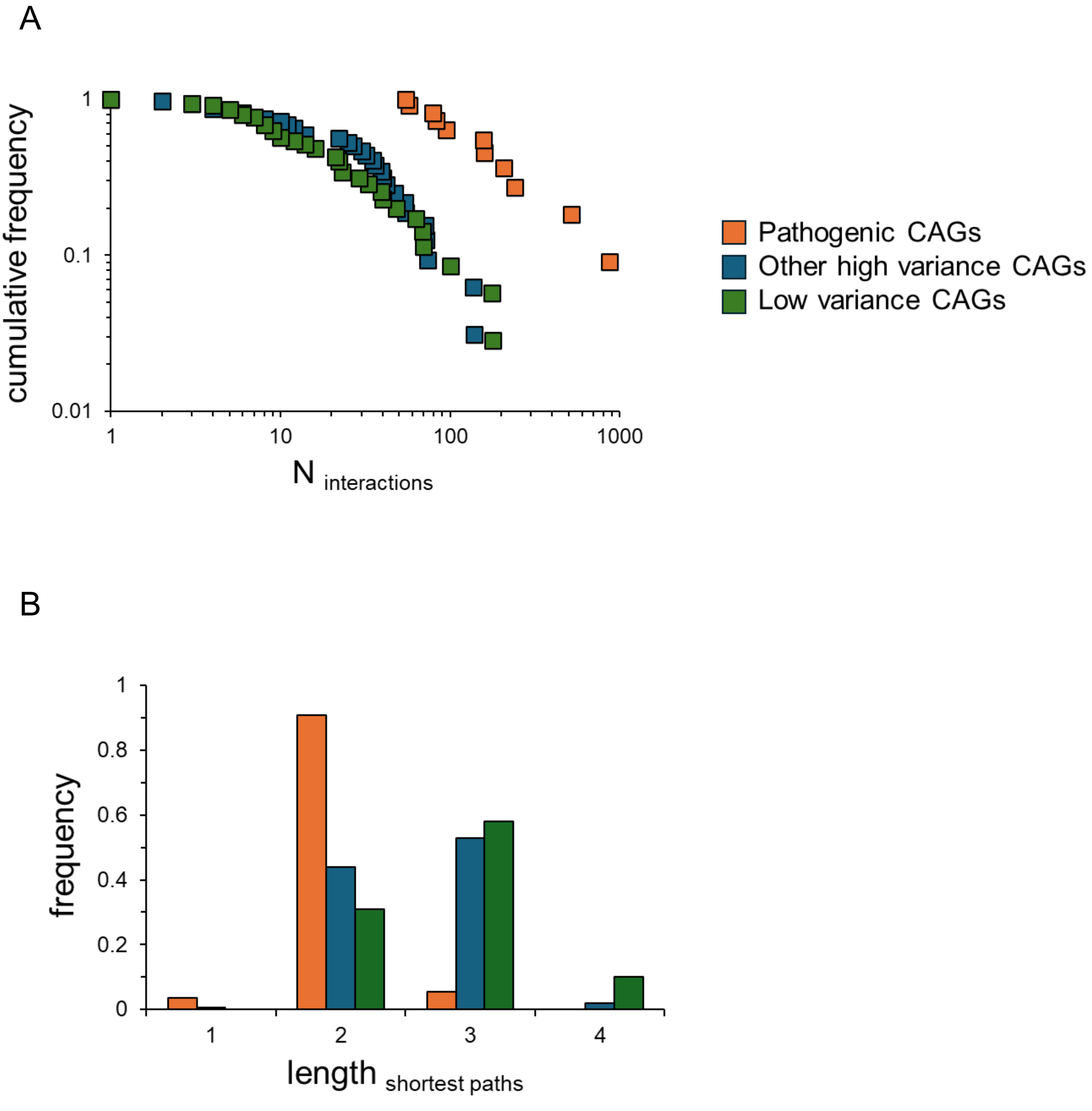

Supplementary Figure 10

A

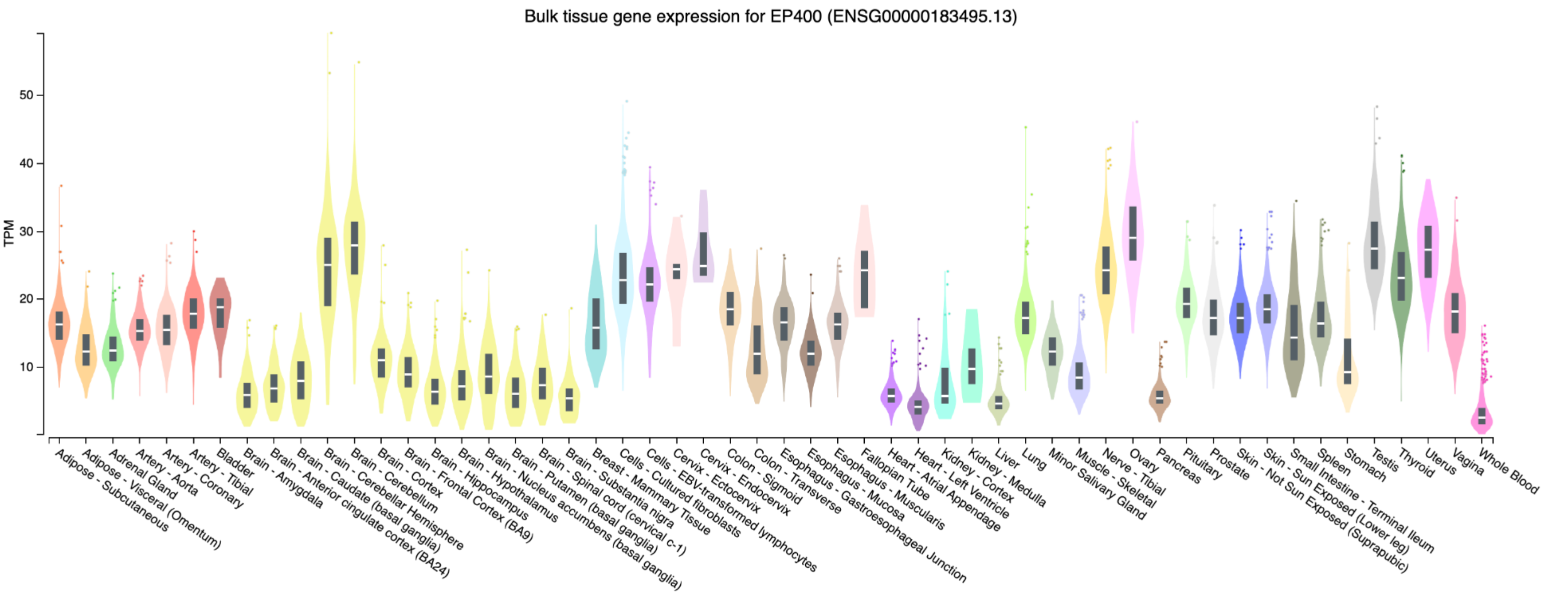

B

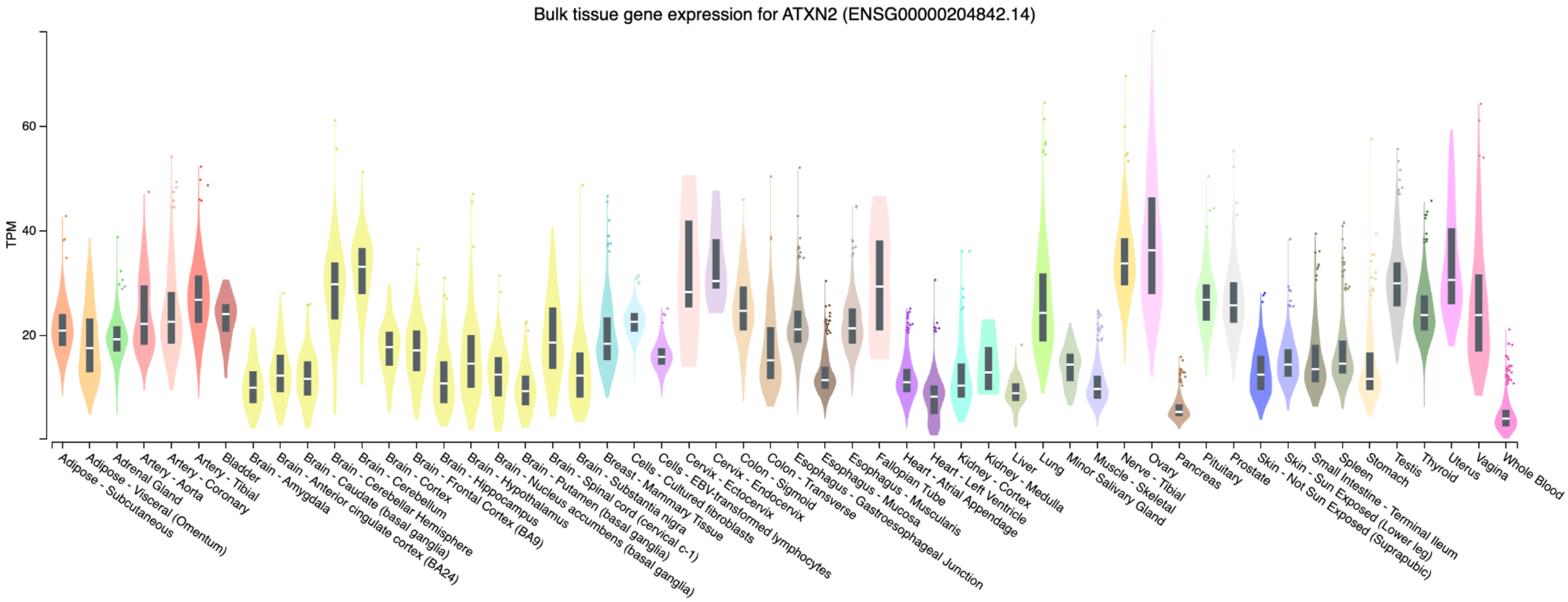

Supplementary Figure 11

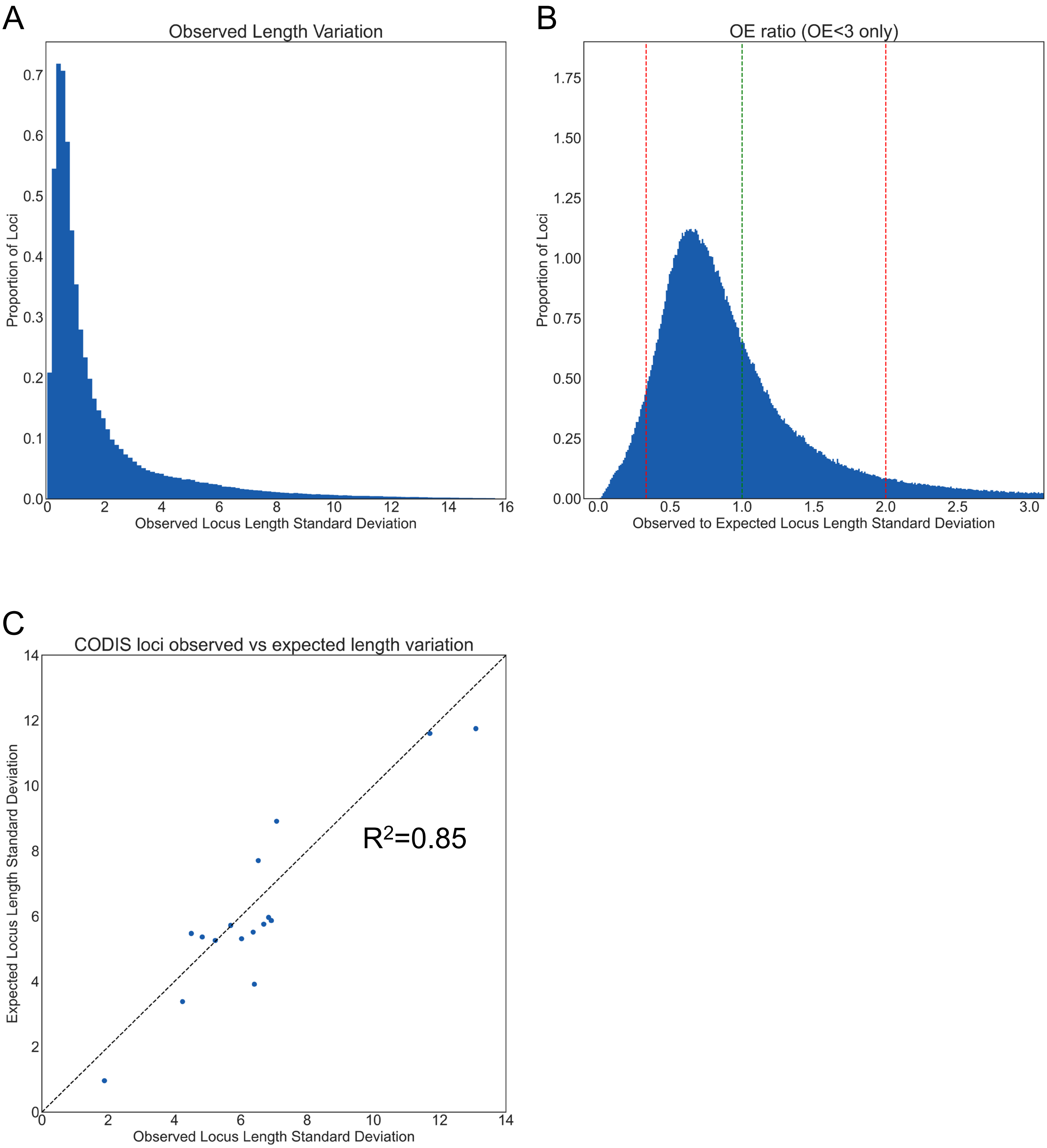

Supplementary Figure 12

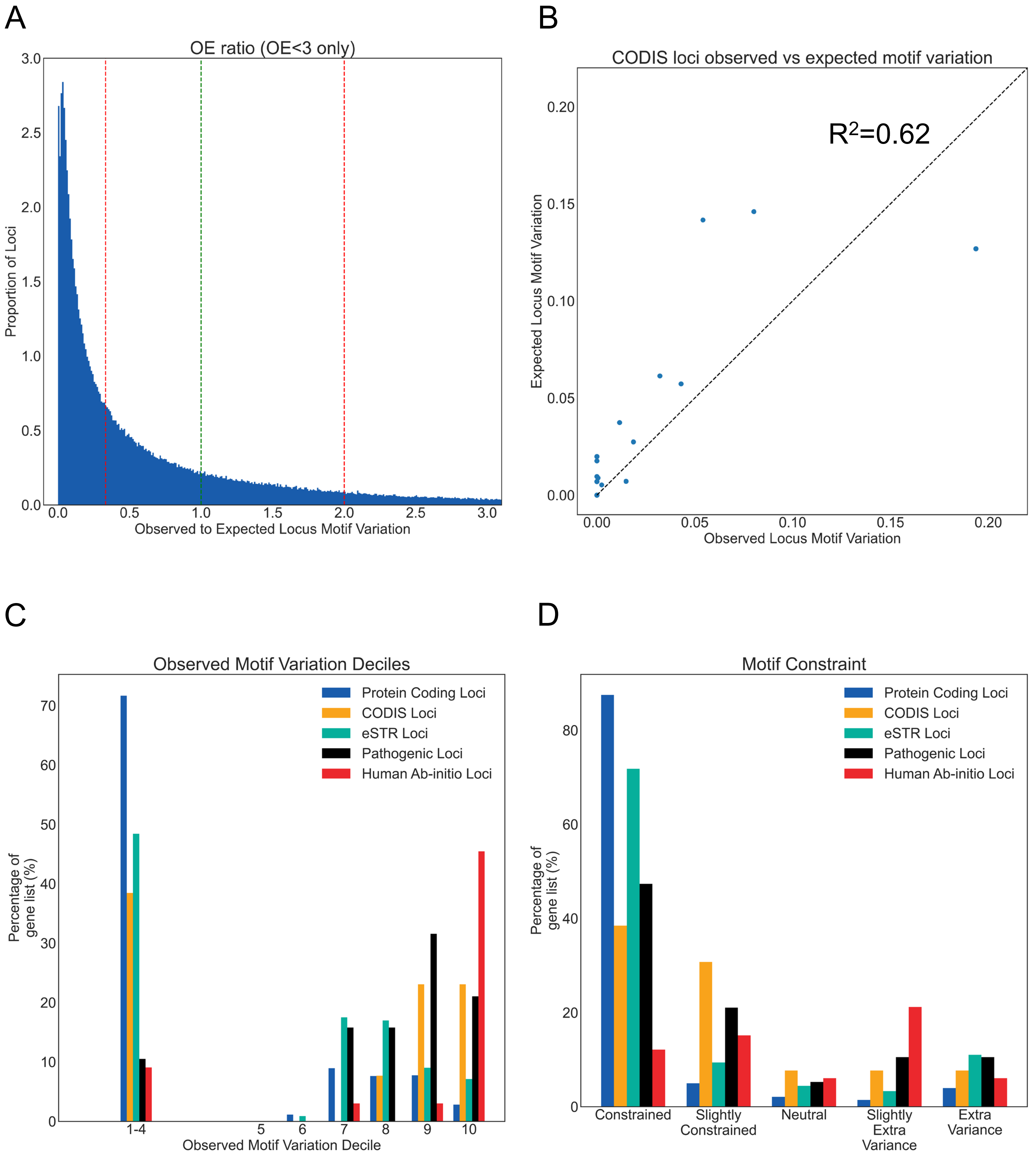

Supplementary Figure 13

A

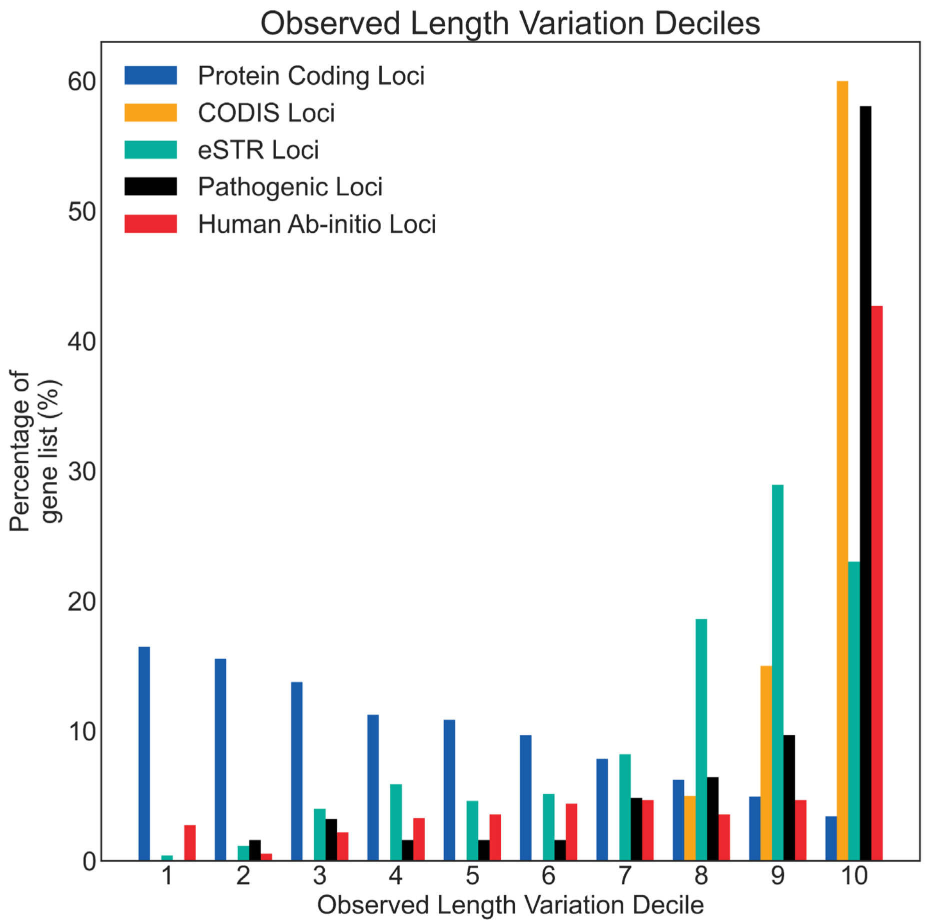

B

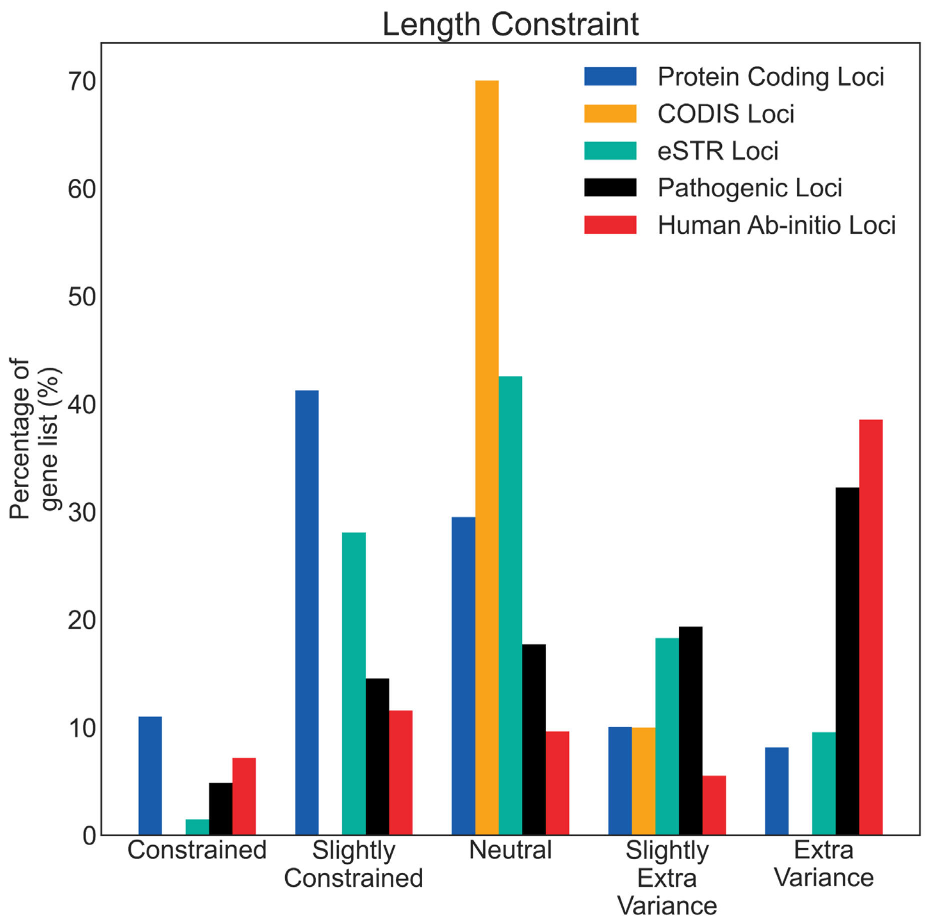

Supplementary Figure 14

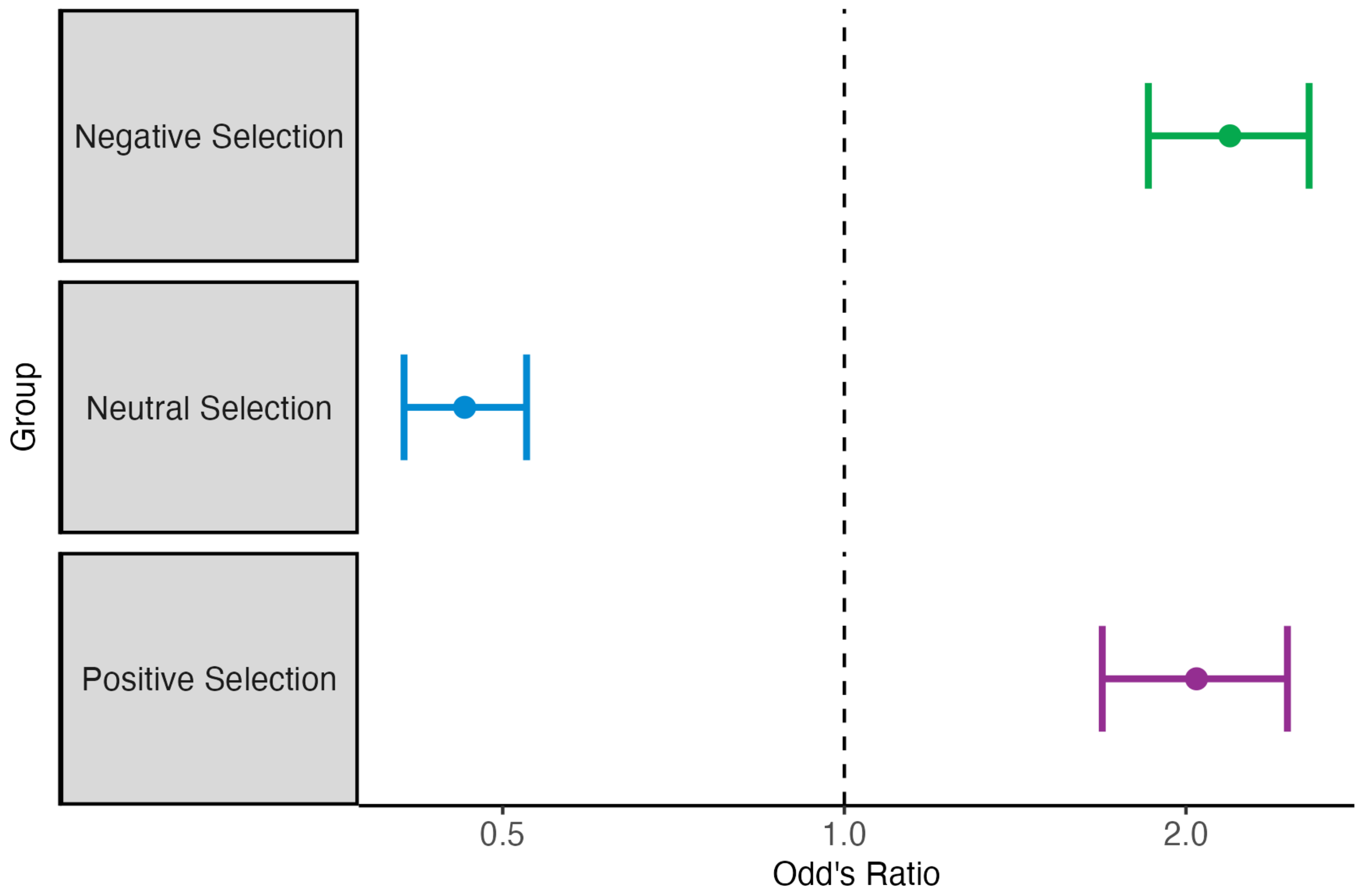

Supplementary Figure 15

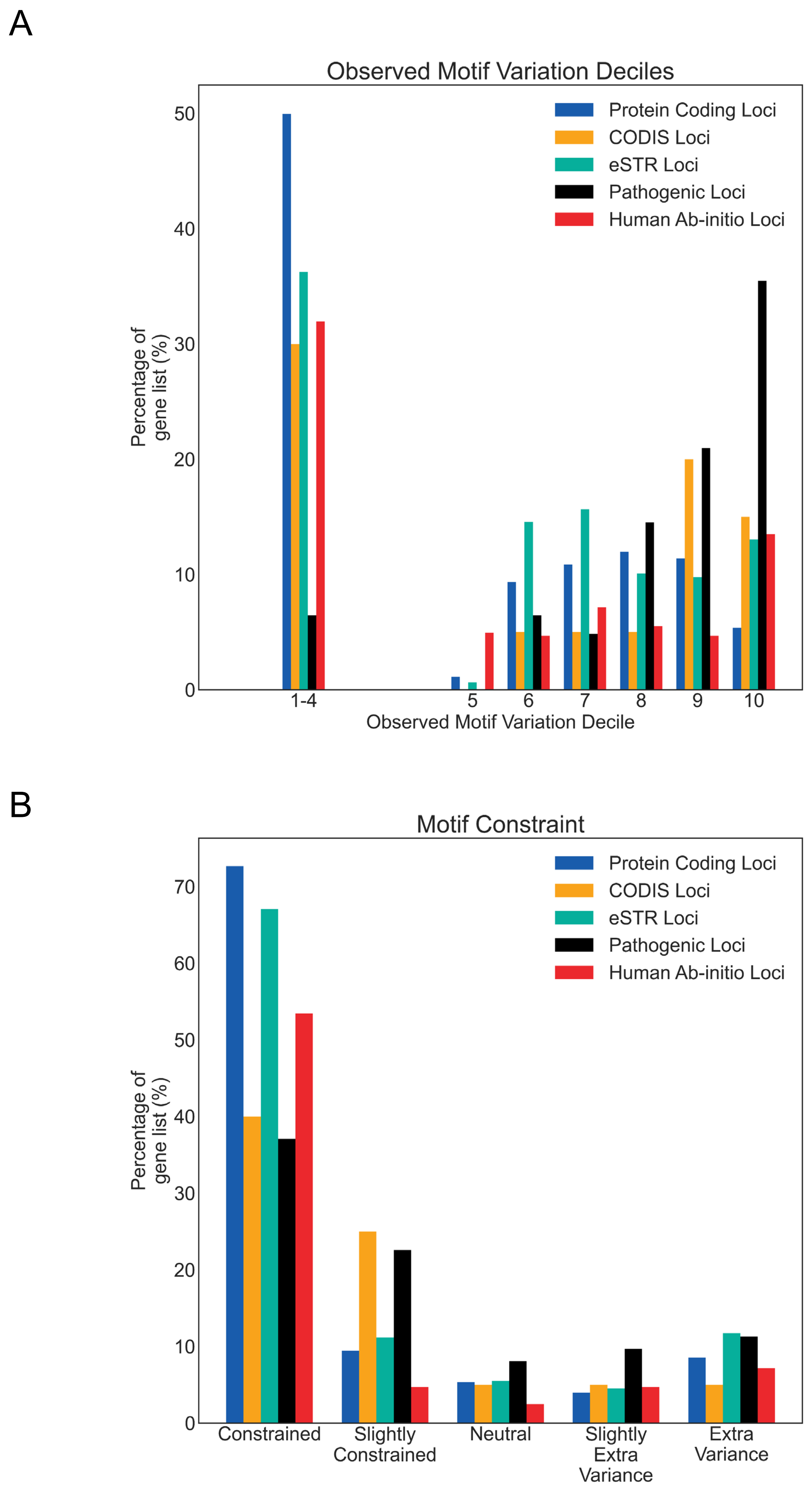

Supplementary Figure 16

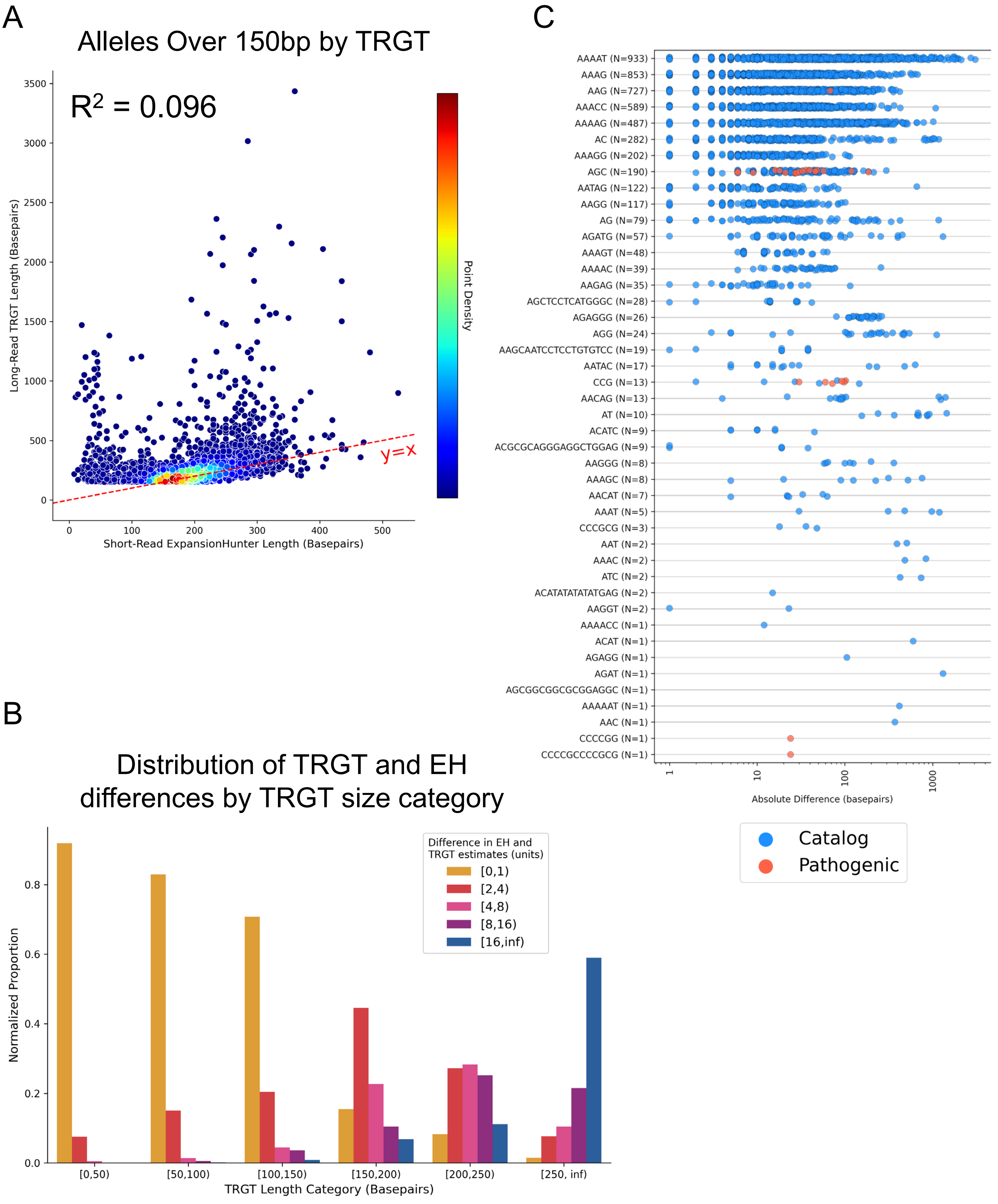

Supplementary Figure 17

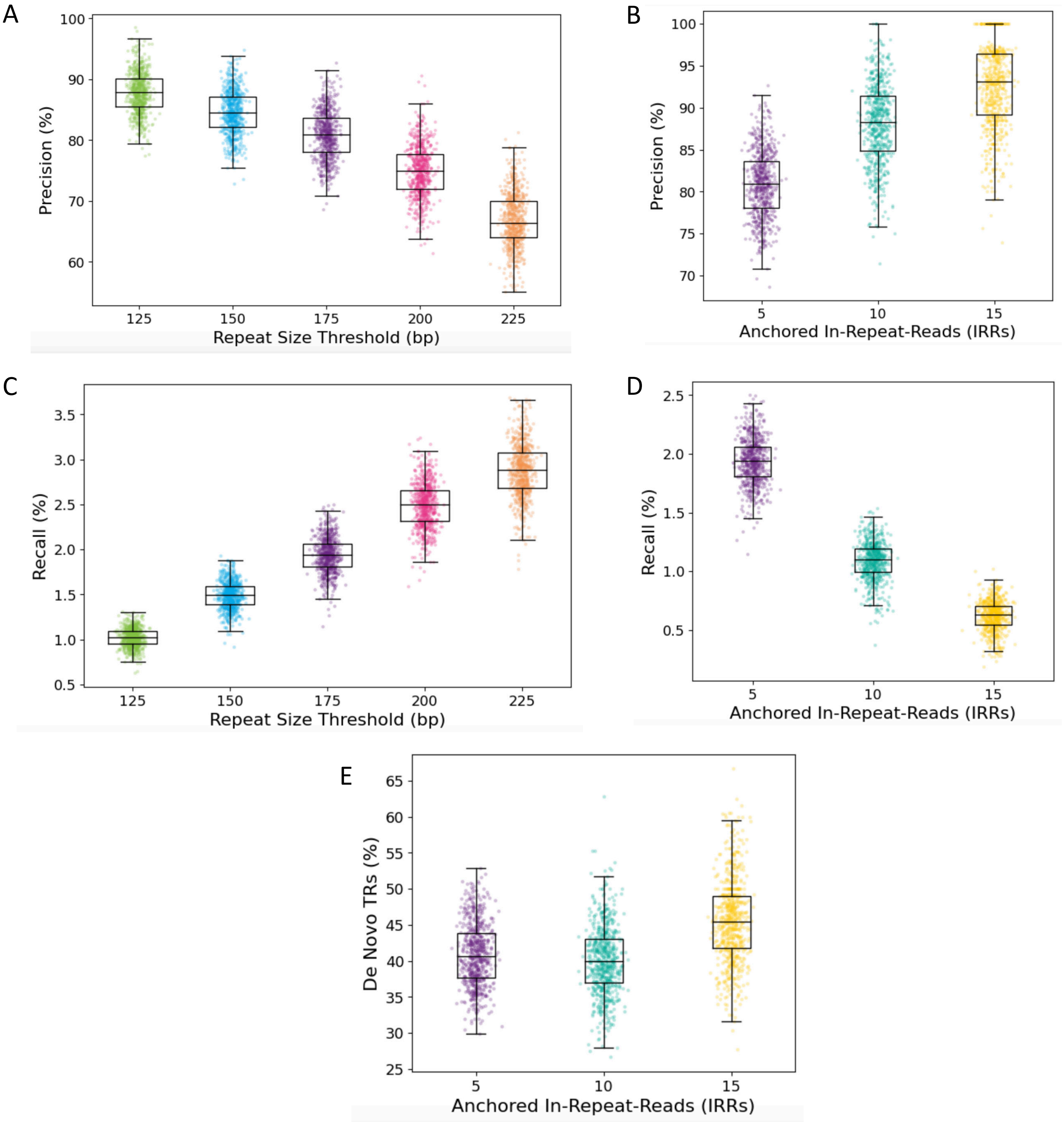

Supplementary Figure 18

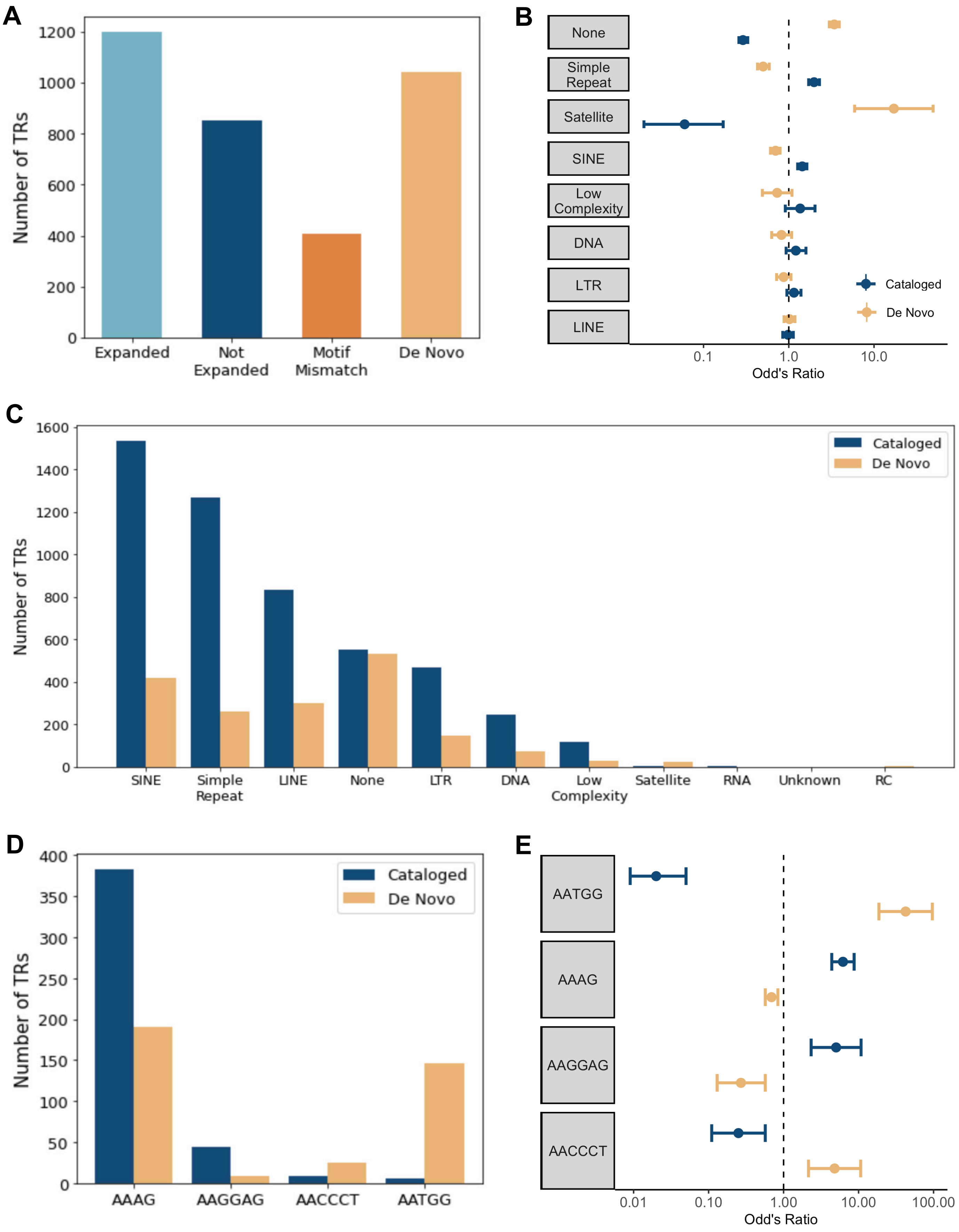

Supplementary Figure 19

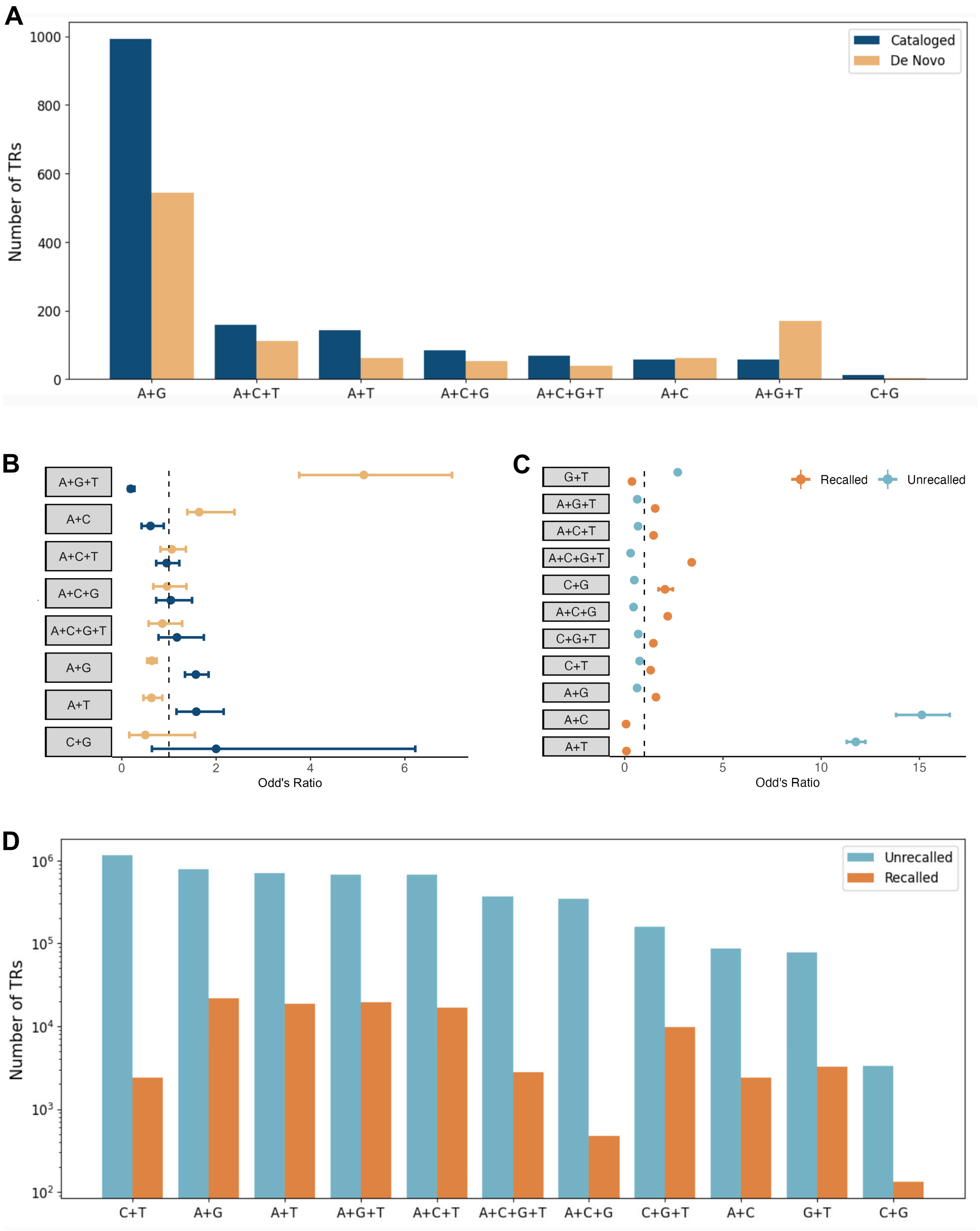

Supplementary Figure 20

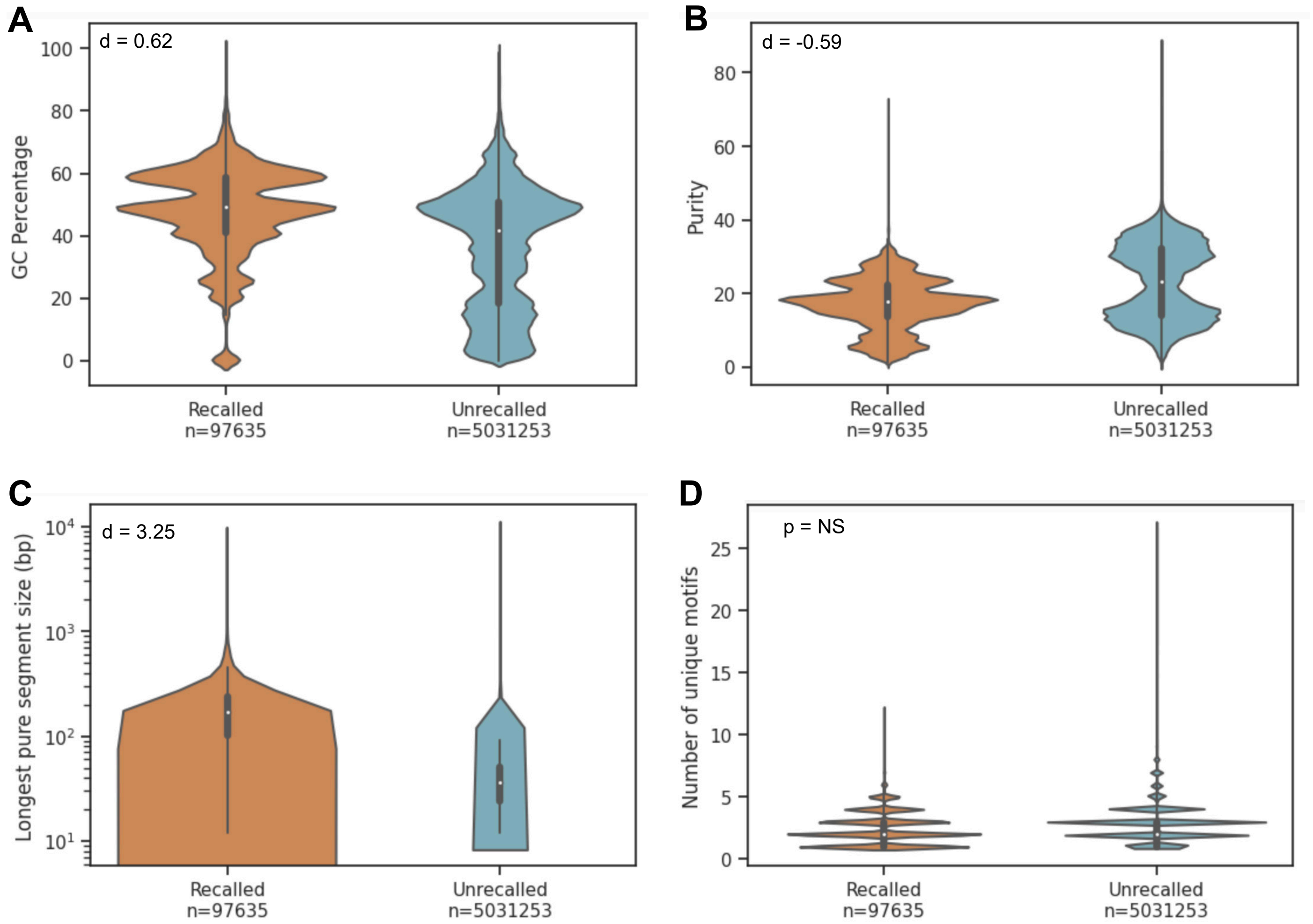

Supplementary Figure 21
